# Supplementary material for: Sources of evidence, pathways, and processes to support disability-inclusive decision-making in low- and middle-income countries: A scoping review
Source: PLOS Glob Public Health. 2025 May 12;5(5):e0004555. doi: 10.1371/journal.pgph.0004555 (PMC12068629; doi:10.1371/journal.pgph.0004555)
Supplement: S2 File — (PDF) [file pgph.0004555.s002.pdf]

Ovid MEDLINE(R) ALL <1946 to August 27, 2023>

1 (national/ or public.mp.) adj2 administration.mp.

2 ((provincial or regulative or public or federal or national or state) adj2 authorit*).mp.

3 (political part* or governing bod* or congress or cabinet or public sector).mp.

4 government organization*.mp.

5 state owned organization*.mp. or Health Maintenance Organizations/

6 Policy Making/

7 Public Health Administration/ or public administration.mp.

8 exp Government/

9 policymaker*.mp. or Public Policy/

10 (government adj2 (agenc* or institutions or officials)).mp.

11 (public adj2 (governance or services)).mp.

12 1 or 2 or 3 or 4 or 5 or 6 or 7 or 8 or 9 or 10 or 11

13 NGO*.tw.

14 ((non governmental or civil society or non profit or philanthropic or independent or community-driven or voluntary or volunteer*) adj2 (organization* or group*)).tw.

15 Voluntary Health Agencies/ or voluntary sector.mp.

16 ((charitable or third-sector or grassroots or community-based) adj2 organization*).tw.

17 (social enterpris* or charit* or non state actor* or advocacy groups*).tw.

18 ((humanitarian or community driven) adj2 organization*).tw.

19 (relief agenc* or OPD or DPO).tw.

20 disabled people organization*.tw.

21 (people with disabilities adj2 (organi* or group*)).tw.

22 13 or 14 or 15 or 16 or 17 or 18 or 19 or 20 or 21

23 12 or 22

24 ((evidence-based or data-driven or research-driven or evidence-informed or fact-based or research-based or empirical or knowledge-based or scientific or rational or objective) adj2 decision-making).mp

25 exp Decision Making/

26 exp evidence-based practice/ or evidence-based medicine/

27 evidence based.tw.

28 exp empirical research/

29 knowledge based.mp.

30 26 or 27 or 28 or 29

31 Policy Making/

32 25 or 31

33 32 and 30

34 33 or 24

35 23 and 34

36 Diversity, Equity, Inclusion/

37 Health Services Accessibility/

38 (disable* or disabilit* or handicapped or (physical* or intellectual* or learning or psychiatric* or sensory or motor or neuromotor or cognitive or mental* or developmental or communication or learning) or (cognitive* or learning or mobility or sensory or visual* or vision or sight or hearing or physical* or mental* or intellectual*)).mp.

39 (impair* or disabilit* or disabl* or handicap*).tw.

40 38 adj 39

41 ((communication or language or speech or learning) adj5 disorder*).mp.

42 ((depression or depressive or anxiety or psychiat*) adj2 (impair* or disabilit* or disabl* or handicap*)).mp

43 ((mental health or schizophreni* or psychos* or psychotic or schizoaffective or schizophreniform or dementia* or alzheimer*) adj2 (impair* or disabilit* or disabl* or handicap*)).mp.

44 ((mental* or emotional* or psychiatric or neurologic*) adj2 (disorder* or ill or illness*)).mp. [

45 (autis* or dyslexi* or Down* syndrome or mongolism or trisomy 21).mp.

46 ((intellectual* or educational* or mental* or psychological* or developmental) adj5 (impair* or retard* or deficien* or disable* or disabili* or handicap* or ill*)).mp.

47 ((hearing or acoustic or ear*) adj5 (loss* or impair* or deficien* or disable* or disabili* or handicap*) or deaf*)).mp.

48 (((visual* or vision or eye* or ocular) adj5 (loss* or impair* or deficien* or disable* or disabili* or handicap*)) or blind*).mp.

49 (cerebral pals* or spina bifida or muscular dystroph* or arthriti* or osteogenesis imperfecta or musculoskeletal abnormalit* or musculo-skeletal abnormalit* or muscular abnormalit* or skeletal abnormalit* or limb abnormalit* or brain injur* or amput* or clubfoot or polio* or paraplegi* or paralys* or paralyz* or hemiplegi* or stroke* or cerebrovascular accident*).mp.

50 (physical* adj5 (impair* or deficien* or disable* or disabili* or handicap*)).mp.

51 exp Disabled Persons/

52 exp Congenital Abnormalities/

53 exp Mental Disorders/

54 exp Learning Disabilities/

55 paralysis/ or paraparesis/ or paraplegia/ or poliomyelitis/ or hearing impairment/ or deafness/

56 Persons With Hearing Impairments/ or Mobility Limitation/ or Amputees/

57 Developmental Disabilities/ or Persons with Mental Disabilities/

58 36 or 37 or 38 or 39 or 40 or 41 or 42 or 43 or 44 or 45 or 46 or 47 or 48 or 49 or 50 or 51 or 52 or 53 or 54 or 55 or 56 or 57

59 35 and 58

60 developing countr*.mp. or Developing Countries/

61 exp Africa/ or exp Asia/ or exp Caribbean Region/ or exp West Indies/ or exp Middle East/ or exp South America/ or exp Latin America/ or exp Central America/

62 (Africa or Asia or Caribbean or West Indies or Middle East or South America or Latin America or Central America).tw.

63 ((developing or less* developed or under developed or underdeveloped or middle income or low* income or underserved or under-served or deprived or poor*) adj (countr* or nation* or population* or world or state*)).mp.

64 ((developing or less* developed or under developed or underdeveloped or middle income or low* income) adj (economy or economies)).tw.

65 (low* adj (gdp or gnp or gross domestic or gross national)).mp.

66 (low adj3 (middle adj3 countr*)).tw.

67 (lmic or lmics or third world or lami countr* or transitional countr*).tw.

68 60 or 61 or 62 or 63 or 64 or 65 or 66 or 67

69 59 and 68

Cochrane Database of Systematic Reviews

Issue 8 of 12, August 2023

#26 (national or public) NEAR/2 administration

#27 (provincial or regulative or public or federal or national or state) NEAR/2 authorit*

#28 ("political party "or "political parties"):ti,ab,kw

#29 ("governing body" or "public sector"): ti,ab,kw

#30 MeSH descriptor: [Health Maintenance Organizations] explode all trees

#31 MeSH descriptor: [Policy Making] explode all trees

#32 "policy making"

#33 (Public Health Administration):ti,ab,kw

#34 (policymaker* or "Public Policy"):ti,ab,kw

#35 (government NEAR/2 (agency or agencies or institutions or officials)):ti,ab,kw

#36 (public NEAR/2 (governance or services)):ti,ab,kw

#37 #26 or #27 or #28 or #29 or #30 or #31 or #32 or #33 or #34 or #35 or #36

#38 (NGO*):ti,ab,kw

#39 non governmental

#40 humanitarian organization*

#41 civil society

#42 non profit

#43 philantropic or "volunteer organization"

#44 voluntary sector

#45 (charitable or third-sector or grassroots or community-based) NEAR/2 organization*

#46 (social enterprise or charit* or non state actor* or advocacy group*)

#47 relief agency or relief agencies

#48 #38 or #39 or #40 or #41 or #42 or #43 or #44 or #45 or #46 or #47

#49 #37 or #48

#50 decision making

#51 policy making

#52 evidence based

#53 empirical research

#54 knowledge based

#55 #50 or #51 or #52 or #53 or #54

#56 #49 and #55

#57 Health Services Accessibility

#58 MeSH descriptor: [Health Services Accessibility] explode all trees

#59 ((disable* or disabilit* or handicap*)):ti,ab,kw

#60 ((communication or language or speech or learning) NEAR/5 disorder*):ti,ab,kw

#61 (depression or depressive or anxiety or psychiatr*) NEAR/2 (impairment or disability or disabled or handicap*)

#62 (mental health or schizophrenia or psychosis or psychotic or schizoaffective or schizophreniform or dementia* or alzheimer):ti,ab,kw

#63 ((mental or emotional or psychiatric or neurologic*) NEAR/2 (disorder* or illness)):ti,ab,kw

#64 (autism or autistic or dyslexia or dyslectic or Down* syndrome or mongolism or trisomy 21):ti,ab,kw

#65 ((hearing or acoustic or ear*) NEAR/5 (loss* or impair* or deficien* or disable* or disabili* or handicap*)):ti,ab,kw

#66 deafness

#67 ((visual* or vision or eye* or ocular) NEAR/5 (loss* or impair* or deficien* or disable* or disabili* or handicap*)):ti,ab,kw

#68 blindness

#69 ((cerebral pals* or spina bifida or muscular dystroph* or arthriti* or osteogenesis imperfecta or musculoskeletal abnormalit* or musculo-skeletal abnormalit* or muscular abnormalit* or skeletal abnormalit* or limb abnormalit* or brain injur* or amput* or clubfoot or polio* or paraplegi* or paralys* or paralyz* or hemiplegi* or stroke* or cerebrovascular accident*)):ti,ab,kw

#70 MeSH descriptor: [Disabled Persons] explode all trees

#71 MeSH descriptor: [Congenital, Hereditary, and Neonatal Diseases and Abnormalities] explode all trees

#72 MeSH descriptor: [Mental Disorders] explode all trees

#73 MeSH descriptor: [Learning Disabilities] explode all trees

#74 #57 or #58 or #59 or #60 or #61 or #62 or #63 or #64 or #65 or #66 or #67 or #68 or #69 or #70 or #71 or #72 or #73

#75 #56 and #74

#76 developing countries

#77 MeSH descriptor: [Developing Countries] explode all trees

#78 Africa or Asia or Caribbean or West Indies or Middle East or South America or Latin America or Central America

#79 (developing or less* developed or under developed or underdeveloped or middle income or low* income or underserved or under-served or deprived or poor*) NEAR (countr* or nation* or population* or world or state*)

#80 (developing or less* developed or under developed or underdeveloped or middle income or low* income) NEAR (economy or economies)

#81 low* NEAR (gdp or gnp or gross domestic or gross national)

#82 (low and middle and income countries)

#83 (lmic or lmics or third world or lami countr* or transitional countr*)

#84 #76 or #77 or #78 or #79 or #80 or #81 or #82 or #83

#85 #75 and #84

Interface - EBSCOhost Research Databases

Database - CINAHL

#	Query
S20	S16 AND S19
S19	S17 OR S18
S18	TX (lami countr* or transitional countr*) OR TX (low* N/1 (gdp or gnp))
S17	TX (developing countries or developing nations or third world or low income countries) OR MH developing countries OR TX LMIC OR TX ((Africa or Asia or Caribbean or West Indies or Middle East or South America or Latin America or Central America)) OR TX ((developing or less* developed or under developed or underdeveloped or middle income or low* income or underserved or under-served or deprived or poor*) N/1 (countr* or nation* or population* or world))
S16	S9 AND S15
S15	S10 OR S11 OR S12 OR S13 OR S14
S14	MH congenital abnormalities OR TX (physical* N3 (impair* or deficien* or disable* or disabili* or handicap*))

S13	TX ((visual* or vision or eye* or ocular) N3 (loss* or impair* or deficien* or disable* or disabili* or handicap*)) OR TX blindness OR TX (cerebral pals* or spina bifida or muscular dystroph* or arthriti* or osteogenesis imperfecta or musculoskeletal abnormalit* or musculo-skeletal abnormalit* or muscular abnormalit* or skeletal abnormalit* or limb abnormalit* or brain injur* or amput* or clubfoot or polio* or paraplegi* or paralys* or paralyz* or hemiplegi* or stroke* or cerebrovascular accident*))
S12	TX (((mental* or emotional* or psychiatric or neurologic*) N2 (disorder* or ill or illness*)) OR TX (autism or autistic or dyslexi* or Down* syndrome or mongolism or trisomy 21)) OR TX ((hearing or acoustic or ear*) N3 (loss* or impair* or deficien* or disable* or disabili* or handicap*)) OR TX (deafness or hearing impairment or deaf or hard of hearing)
S11	TX (((communication or language or speech or learning) N4 disorder*) OR TX ((depression or depressive or anxiety or psychiat*) N2 (impair* or disabilit* or disabl* or handicap*)) OR TX ((mental health or schizophreni* or psychos* or psychotic or schizoaffective or schizophreniform or dementia* or alzheimer*))
S10	MH health services accessibility OR MH disabled persons OR TX (disabled persons or people with disabilities) OR TX (disability or disabilities or disabled or impairment or impaired or special or special needs)
S9	S5 AND S8
S8	S6 OR S7
S7	MH decision making OR MH policy making OR TX (decision making or decision-making or decision making process or decision-making process)

S6	TX evidence-based or data-driven or research-driven or evidence-informed or fact-based or research-based or empirical or knowledge-based or scientific or rational or objective) N2 decision-making
S5	S1 OR S2 OR S3 OR S4
S4	TX ((social enterpris* or charit* or non state actor* or advocacy group*)) OR TX ((humanitarian or community-driven) N2 organization*) OR TX ((relief agenc* or OPD or DPO))
S3	TX (ngo or non-governmental organization or nonprofit or non-profit or nongovernmental organization) OR TX ((philanthropic or independent or community-driven or voluntary or volunteer*) N2 (organization* or group*)) OR TX ((charitable or third-sector or grassroots or community-based) N2 organization*)
S2	TX ((political part* or governing bod* or public sector)) OR TX (government organization*OR Health Organization*) OR TX public health administration OR TX (policy makers or decision makers) OR TX (government N2 (agenc* OR institutions OR officials)) OR TX (public N2 (governance OR services))
S1	TX ((national or public) N2 administration) OR TX ((provincial or regulative or public or federal or national or state) N2 authorit*)

Interface - EBSCOhost Research Databases

Database - APA PsycInfo

Database - ERIC

#	**Query**

S20	S16 AND S19
S19	S17 OR S18
S18	TX (lami countr* or transitional countr*) OR TX (low* N/1 (gdp or gnp))
S17	TX (developing countries or developing nations or third world or low income countries) OR developing countries OR TX LMIC OR TX ((Africa or Asia or Caribbean or West Indies or Middle East or South America or Latin America or Central America)) OR TX ((developing or less* developed or under developed or underdeveloped or middle income or low* income or underserved or under-served or deprived or poor*) N/1 (countr* or nation* or population* or world))
S16	S9 AND S15
S15	S10 OR S11 OR S12 OR S13 OR S14
S14	congenital abnormalities OR TX (physical* N3 (impair* or deficien* or disable* or disabili* or handicap*))
S13	TX ((visual* or vision or eye* or ocular) N3 (loss* or impair* or deficien* or disable* or disabili* or handicap*)) OR TX blindness OR TX (cerebral pals* or spina bifida or muscular dystroph* or arthriti* or osteogenesis imperfecta or musculoskeletal abnormalit* or musculo-skeletal abnormalit* or muscular abnormalit* or skeletal abnormalit* or limb abnormalit* or brain injur* or amput* or clubfoot or polio* or paraplegi* or paralys* or paralyz* or hemiplegi* or stroke* or cerebrovascular accident*))
S12	TX (((mental* or emotional* or psychiatric or neurologic*) N2 (disorder* or ill or illness*)) OR TX (autism or autistic or dyslexi* or Down* syndrome or mongolism or trisomy 21)) OR TX ((hearing or acoustic or ear*) N3 (loss* or impair* or deficien*

	or disable* or disabili* or handicap*)) OR TX (deafness or hearing impairment or deaf or hard of hearing)
S11	TX (((communication or language or speech or learning) N4 disorder*) OR TX ((depression or depressive or anxiety or psychiat*) N2 (impair* or disabilit* or disabl* or handicap*)) OR TX ((mental health or schizophreni* or psychos* or psychotic or schizoaffective or schizophreniform or dementia* or alzheimer*))
S10	health services accessibility OR disabled persons OR TX (disabled persons or people with disabilities) OR TX (disability or disabilities or disabled or impairment or impaired or special or special needs)
S9	S5 AND S8
S8	S6 OR S7
S7	decision making OR policy making OR TX (decision making or decision-making or decision making process or decision-making process)
S6	TX evidence-based or data-driven or research-driven or evidence-informed or fact-based or research-based or empirical or knowledge-based or scientific or rational or objective) N2 decision-making
S5	S1 OR S2 OR S3 OR S4
S4	TX ((social enterpris* or charit* or non state actor* or advocacy group*)) OR TX ((humanitarian or community-driven) N2 organization*) OR TX ((relief agenc* or OPD or DPO))
S3	TX (ngo or non-governmental organization or nonprofit or non-profit or nongovernmental organization) OR TX ((philanthropic or independent or community-

	driven or voluntary or volunteer*) N2 (organization* or group*)) OR TX ((charitable or third-sector or grassroots or community-based) N2 organization*)
S2	TX ((political part* or governing bod* or public sector)) OR TX (government organization*OR Health Organization*) OR TX public health administration OR TX (policy makers or decision makers) OR TX (government N2 (agenc* OR institutions OR officials)) OR TX (public N2 (governance OR services))
S1	TX ((national or public) N2 administration) OR TX ((provincial or regulative or public or federal or national or state) N2 authorit*)

Embase 1947-Present, updated daily

1 ((national or public) adj2 administration).mp.

2 ((provincial or regulative or public or federal or national or state) adj2 authorit*).mp.

3 (political part* or governing bod* or public sector).mp.

4 government/ and organization/

5 health maintenance organization/

6 policy making.mp.

7 public health administration.mp. or public health service/

8 exp government/

9 (government adj2 (agenc* or institutions or officials)).mp.

10 (public adj2 (governance or services)).mp.

11 1 or 2 or 3 or 4 or 5 or 6 or 7 or 8 or 9 or 10

12 NGO*.tw.

13 ((non governmental or civil society or non profit or philanthropic or independent or community-driven or voluntary or volunteer*) adj2 (organization* or group*)).tw.

14 voluntary sector.mp.

15 ((charitable or third-sector or grassroots or community-based) adj2 organization*).tw.

16 (social enterpris* or charit* or non state actor* or advocacy groups*).tw.

17 ((humanitarian or community driven) adj2 organization*).tw.

18 (relief agenc* or OPD or DPO).tw.

19 12 or 13 or 14 or 15 or 16 or 17 or 18

20 11 or 19

21 ((evidence-based or data-driven or research-driven or evidence-informed or fact-based or research-based or empirical or knowledge-based or scientific or rational or objective) adj2 decision-making).mp.

22 exp decision making/

23 evidence based practice/

24 policy making.mp.

25 medical decision making/

26 21 or 22 or 23 or 24 or 25

27 20 and 26

28 health services accessibility.mp. or health care access/

29 (impair* or disabilit* or disabl* or handicap*).tw.

30 ((communication or language or speech or learning) adj3 disorder*).mp.

31 ((depression or depressive or anxiety or psychiat*) adj2 (impair* or disabilit* or disabl* or handicap*)).mp.

32 (mental health or schizophreni* or psychos* or psychotic or schizoaffective or schizophreniform or dementia* or alzheimer*).mp.

33 ((mental* or emotional* or psychiatric or neurologic*) adj2 (disorder* or ill or illness*)).mp.

34 (autis* or dyslexi* or Down* syndrome or mongolism or trisomy 21).mp.

35 ((intellectual* or educational* or mental* or psychological* or developmental) adj5 (impair* or retard* or deficien* or disable* or disabili* or handicap* or ill*)).mp.

36 ((hearing or acoustic or ear*) adj5 (loss* or impair* or deficien* or disable* or disabili* or handicap*)).mp.

37 hearing impairment/

38 deafness.tw.

39 ((visual* or vision or eye* or ocular) adj3 (loss* or impair* or deficien* or disable* or disabili* or handicap*)).mp.

40 *blindness/

41 (cerebral pals* or spina bifida or muscular dystroph* or arthriti* or osteogenesis imperfecta or musculoskeletal abnormalit* or musculo-skeletal abnormalit* or muscular abnormalit* or skeletal abnormalit* or limb abnormalit* or brain injur* or amput* or clubfoot or polio* or paraplegi* or paralys* or paralyz* or hemiplegi* or stroke*).mp.

42 congenital disorder/

43 28 or 29 or 30 or 31 or 32 or 33 or 34 or 35 or 36 or 37 or 38 or 39 or 40 or 41 or 42

44 (physical* adj5 (impair* or deficien* or disable* or disabili* or handicap*)).mp.

45 43 or 44

46 27 and 45

47 exp developing country/

48 developing countries.mp.

49 "South and Central America"/

50 Caribbean/

51 Caribbean Islands/

52 "South and Central America"/

53 exp Africa/ or exp Asia/ or exp West Indies/

54 Pacific islands/

55 exp Middle East/

56 (Africa or Asia or Caribbean or West Indies or Middle East or South America or Latin America or Central America).tw.

57 ((developing or less* developed or under developed or underdeveloped or middle income or low* income or underserved or under-served or deprived or poor*) adj (countr* or nation? or population? or world or state*)).mp.

58 ((developing or less* developed or under developed or underdeveloped or middle income or low* income) adj (economy or economies)).tw.

59 (low* adj (gdp or gnp or gross domestic or gross national)).mp.

60 (lmic or lmics or third world or lami countr* or transitional countr*).tw.

61 (low adj3 (middle adj3 countr*)).tw.

62 47 or 48 or 49 or 50 or 51 or 52 or 53 or 54 or 55 or 56 or 57 or 58 or 59 or 60 or 61

63 46 and 62

Scopus (Elseviers)

((((TITLE-ABS-KEY ((impair* OR disabilit* OR disabl* OR handicap*))) OR (TITLE-ABS-KEY ((mental AND health OR schizophreni* OR psychos* OR psychotic OR schizoaffective OR schizophreniform OR dementia* OR alzheimer*))) OR (TITLE-ABS-KEY ((autis* OR dyslexi* OR down* AND syndrome OR mongolism OR trisomy 21))) OR (TITLE-ABS-KEY (hearing AND impairment OR deafness OR visual AND impairment OR blindness)) OR (TITLE-ABS-KEY (congenital AND disorder*)) OR (TITLE-ABS-KEY (physical AND impairment OR physical AND disability))) AND (TITLE-ABS-KEY (decision AND making OR policy AND making))) AND (TITLE-ABS-KEY (government* OR "public sector" OR "public services" OR "public administration" OR ngo* OR "voluntary organization*" OR "charitable organization" OR "non-government*" OR "non profit" OR "advocacy group*"))) AND ((TITLE-ABS-KEY (developing AND countries)) OR (TITLE-ABS-KEY (low AND middle AND income AND countries)) OR (TITLE-ABS-KEY (lmic)) OR (TITLE-ABS-KEY ("lami countr* " OR "transitional countr*")) OR (TITLE-ABS-KEY (low* AND (gdp OR gnp OR gross AND domestic OR gross AND national)))))

Web of Science Core Collection Editions: WOS.SSCI

Search Query
#1 (national or public) AND administration (Topic) OR (provincial or regulative or public or federal or national or state) AND authorit* (Topic) OR (political part* or governing bod* or public sector) OR (government organization*OR Health Organization*) OR (public health administration) (Topic) OR (policy

makers or decision makers) OR ((government AND (agenc* OR institutions OR officials))) (Topic) Editions: WOS.SSCI
#2 (ngo or non-governmental organization or nonprofit or non-profit or nongovernmental organization) (Topic) OR (philanthropic or independent or community-driven or voluntary or volunteer*) AND (organization* or group*) (Topic) OR (charitable or third-sector or grassroots or community-based) AND organization* (Topic) Editions: WOS.SSCI
#3 #1 OR #2 Editions: WOS.SSCI
#4 (social enterpris* or charit* or non state actor* or advocacy group*) (Topic) OR (humanitarian or community-driven) AND organization* (Topic) OR (relief agenc*) or OPD or DPO (Topic) Editions: WOS.SSCI
#5 #3 OR #4 Editions: WOS.SSCI
#6 (evidence-based or data-driven or research-driven or evidence-informed or fact-based or research-based or empirical or knowledge-based or scientific or rational or objective) AND decision-making (Topic) OR decision making or decision-making or decision making process (Topic) Editions: WOS.SSCI
#7 #5 AND #6 Editions: WOS.SSCI
#8 (health services accessibility) OR disabled persons OR (people with disabilities) OR (disability or disabilities or disabled or impairment or impaired or special or special needs) (Topic) Editions: WOS.SSCI
#9 (communication or language or speech or learning) AND disorder* (Topic) OR (depression or depressive or anxiety or psychiat*) AND (impair* or disabilit* or disabl* or handicap*) (Topic) OR (mental health or schizophreni* or psychos* or

psychotic or schizoaffective or schizophreniform or dementia* or alzheimer*) (Topic) Editions: WOS.SSCI
#10 autism or autistic or dyslexi* or Down* syndrome or mongolism or trisomy 21 (Topic) OR deafness or hearing impairment or deaf (Topic) OR blindness OR cerebral pals* or spina bifida or muscular dystroph* or arthriti* or osteogenesis imperfecta or musculoskeletal abnormalit* or musculo-skeletal abnormalit* or muscular abnormalit* or skeletal abnormalit* or limb abnormalit* or brain injur* or amput* or clubfoot or polio* or paraplegi* or paralys* or paralyz* or hemiplegi* or stroke* (Topic) Editions: WOS.SSCI
#11 congenital abnormalities (Topic) Editions: WOS.SSCI
#12 #8 OR #9 OR #10 OR #11 Editions: WOS.SSCI
#13 #12 AND #7 Editions: WOS.SSCI
#14 (developing countries or developing nations or third world or low income countries OR LMIC) (Topic) OR (Africa or Asia or Caribbean or West Indies or Middle East or South America or Latin America or Central America) (Topic) OR (developing or less* developed or under developed or underdeveloped or middle income or low* income or underserved or under-served or deprived or poor*) AND (countr* or nation* or population* or world) (Topic) Editions: WOS.SSCI
#15 (lami countr*) or (transitional countr*) (Topic) OR low* AND (gdp or gnp) (Topic) Editions: WOS.SSCI
#16 #14 OR #15 Editions: WOS.SSCI
#17 #16 AND #13 Editions: WOS.SSCI
