# Supplementary material for: Sources of evidence, pathways, and processes to support disability-inclusive decision-making in low- and middle-income countries: A scoping review
Source: PLOS Glob Public Health. 2025 May 12;5(5):e0004555. doi: 10.1371/journal.pgph.0004555 (PMC12068629; doi:10.1371/journal.pgph.0004555)
Supplement: S3 File — (DOCX) [file pgph.0004555.s003.docx]

**Google scholar hand search:**

**Years: 1990 - 29 November 2023**

| **Search string number** | **Key word 1** | **Key word 2** | **Key word 3** |
| --- | --- | --- | --- |
| 1 | disability | evidence-based | LMIC |
| 2 | disability | decision-making | developing country |
| 3 | low-income | disability | policy |
| 4 | evidence | disability | program |
| 5 | LMIC | decision-maker | disability |
| 6 | policy | Asia | disability |
| 7 | impairment | policy | LMIC |
| 8 | evidence-based | mental health | LMIC |
| 9 | Africa | decision-making | disabled |
| 10 | policy | people with disabilities | LMIC |
| 11 | blind | decision-making | LMIC |
| 12 | LMIC | deaf | evidence-informed |
| 13 | LMIC | evidence-based | cerebral palsy |
| 14 | stroke | decision-making | LMIC |
| 15 | autism | policy | LMIC |
| 16 | disability | evidence-based | global south |
| 17 | disability | decision-making | latin america |
| 18 | low-income | disability | stakeholder |
| 19 | evidence-based | decision-making | disability |
| 20 | LMIC | evidence-informed | mental health |

| **SEARCH ENGINE** | **SEARCH TERMS YEILDING MOST RESULTS** |
| --- | --- |
| **Google scholar** |  |
|  | evidence based policy making disability |
| **Google** |  |
|  | impairment policy LMIC |
|  | policy Asia disability |
| **Organisation/agency websites** |  |
| Save the Children | disability |
| International Disability Alliance | report |
| World Bank | disab* |
| CBM | evidence-based disability policy |
|  | evidence-based decision-making disability |

**Grey literature search strings that yielded the most/only results per search engine: Years: 1990 - 14 December 2023**
