# Supplementary material for: Sources of evidence, pathways, and processes to support disability-inclusive decision-making in low- and middle-income countries: A scoping review
Source: PLOS Glob Public Health. 2025 May 12;5(5):e0004555. doi: 10.1371/journal.pgph.0004555 (PMC12068629; doi:10.1371/journal.pgph.0004555)
Supplement: Attachment — Submitted filename: PGPH-D-24-02548_reviewed_SN.pdf [file pgph.0004555.s005.pdf]

// PLOS Global Public Health

Sources of evidence, pathways, and processes to support disability-inclusive decision-making in low- and middle-income countries: A scoping review

--Manuscript Draft--

**Manuscript Number:**	PGPH-D-24-02548
**Article Type:**	Research Article
**Full Title:**	Sources of evidence, pathways, and processes to support disability-inclusive decision-making in low- and middle-income countries: A scoping review
**Short Title:**	Sources of evidence, pathways, and processes in LMIC disability-inclusive decision-making
**Corresponding Author:**	Ebruphiyo Ruth Useh, MPH<br>Stellenbosch University - Tygerberg Campus: Stellenbosch University Faculty of Medicine and Health Sciences<br>Cape Town, Western Cape SOUTH AFRICA
**Order of Authors:**	Ebruphiyo Ruth Useh, MPH
	Zara Trafford, PhD
	Prince Changole, PhD
	Xanthe Hunt, PhD
**Keywords:**	evidence-based practice; political economy; disability-inclusive development; policy; programme development; Low- and middle-income countries
**Abstract:**	Widespread failure to sufficiently account for disability in policy and programming drives the exclusion of people with disabilities. These oversights are especially marked in low- and middle-income countries (LMICs), where 80% of the world's 1.3 billion people with disabilities reside. To support more disability-inclusive policy-making and programming in LMICs, it is important to understand how decision-makers think about disability in their work, including which sources of evidence, processes, and pathways they rely on to make policy and programming decisions about disability, and what influences (aside from evidence) shape their decision-making.<br>We conducted a scoping review of literature concerning the use of evidence in governmental and non-governmental decision-making around disability-inclusion in LMICs. We conducted systematic searches of databases of peer-reviewed literature and used thorough hand searches to gather grey literature. Documents were eligible if they focused on key governmental and non-governmental stakeholders; disability-inclusive or disability-related decision-making in mainstream and targeted planning, policymaking, programming, and evaluations; and were based on data from LMICs. All literature was double screened and extracted according to a standardised extraction sheet by four reviewers, working in pairs.<br>We included 38 papers. The majority were focused on mental health, and the rest on disability broadly, or on specific impairment groups. Sources of evidence cited were highly variable and included both empirical and experiential evidence. Barriers and facilitators to using evidence varied by evidence source. Other notable influences on decision-making included government legislation, power dynamics and involvement; stakeholder relationships; the local landscape; funding; and attitudes towards disability.<br>This work highlights the barriers to, and enablers of, evidence utilisation in relation to disability, which can be targets of interventions. Moreover, this review suggests that supporting evidence-based decision-making in relation to disability in LMICs necessitates engagement with varied framings of evidence and influences on decision-making outside of evidence.
**Additional Information:**	
**Question**	**Response**
**Financial Disclosure**	This scoping review was funded in full by Wellspring Philanthropies (grant ID: 17737; PI: XH). The funders had no role in study design, data collection and analysis, the decision to publish, nor the preparation of this manuscript. Funder URL:

Powered by Editorial Manager® and ProduXion Manager® from Aries Systems Corporation

Enter a financial disclosure statement that describes the sources of funding for the work included in this submission and the role the funder(s) played. This includes grants and any commercial funding of the work or authors.<br><br>This statement will be typeset if the manuscript is accepted for publication.<br><br>*Please review the submission guidelines and the instructions link below for detailed requirements and guidance.*	https://wpfund.org/
**Competing Interests**<br><br>On behalf of all authors, disclose any competing interests that could be perceived to bias this work.<br><br>This statement will be typeset if the manuscript is accepted for publication.<br><br>*Please review the instructions link below and PLOS Global Public Health's competing interests policy to determine what information must be disclosed at submission.*	The authors declare no competing interests.
**Data Availability**<br><br>Before publication, Authors are required to make fully available and without restriction all data underlying their findings. Please see our PLOS Data Policy page for detailed information on this policy.<br><br>A **Data Availability Statement**, detailing where the data can be accessed, is required at first submission. Insert your Data Availability Statement in the box below.	The data underlying this research is available in our data repository on the Open Science Framework (OSF) at https://osf.io/xru2v/).

Please see the data reporting section of our submission guidelines for instructions on what you need to include in your Data Availability Statement.<br><br>This statement will be typeset if the manuscript is accepted for publication.<br><br>*PLOS allows rare exemptions to address legal and ethical concerns. If you have legal or ethical restrictions, please use the box below to detail these in full sentences for the Journal team to consider.*	

Powered by Editorial Manager® and ProduXion Manager® from Aries Systems Corporation

Manuscript

Click here to access/download;Manuscript;Disability-inclusive decision-making in LMICs_ScR.docx

Sources of evidence, pathways, and processes to support disability-inclusive decision-making in low- and middle-income countries: A scoping review

Authors:

Ebruphiyo Ruth Useh[1]*, Zara Trafford[1], Prince Changole[1], and Xanthe Hunt [1, 2]

[1] Institute for Life Course Health Research, Department of Global Health, Stellenbosch University, Cape Town, Western Cape, South Africa

[2] Africa Health Research Institute (AHRI), Somkhele, KwaZulu-Natal, South Africa

*Corresponding author

E-mail: Useh@sun.ac.za

Abstract

Widespread failure to sufficiently account for disability in policy and programming drives the exclusion of people with disabilities. These oversights are especially marked in low- and middle-income countries (LMICs), where 80% of the world's 1.3 billion people with disabilities reside. To support more disability-inclusive policy-making and programming in LMICs, it is important to understand how decision-makers think about disability in their work, including which sources of evidence, processes, and pathways they rely on to make policy and programming decisions about disability, and what influences (aside from evidence) shape their decision-making.

We conducted a scoping review of literature concerning the use of evidence in governmental and non-governmental decision-making around disability-inclusion in LMICs. We conducted systematic searches of databases of peer-reviewed literature and used thorough hand searches to gather grey literature. Documents were eligible if they focused on key governmental and non-governmental stakeholders; disability-inclusive or disability-related decision-making in mainstream and targeted planning, policymaking, programming, and evaluations; and were based on data from LMICs. All literature was double screened and extracted according to a standardised extraction sheet by four reviewers, working in pairs.

We included 38 papers. The majority were focused on mental health, and the rest on disability broadly, or on specific impairment groups. Sources of evidence cited were highly variable and included both empirical and experiential evidence. Barriers and facilitators to using evidence varied by evidence source. Other notable influences on decision-making included government legislation, power dynamics and involvement; stakeholder relationships; the local landscape; funding; and attitudes towards disability. This work highlights the barriers to, and enablers of, evidence utilisation in relation to disability, which can be targets of interventions. Moreover, this review suggests that supporting evidence-

based decision-making in relation to disability in LMICs necessitates engagement with varied framings of evidence and influences on decision-making outside of evidence.

Introduction

Although about 1.3 billion people worldwide have a disability, individuals with disabilities consistently experience poorer access to services, and worse outcomes, in education, health, employment, and community participation [1,2]. At least in part, this results from the continued neglect of disability inclusion in policies, legislation, services and interventions in planning and programming globally [3–5].

Over the last two decades, attention to ‘evidence-based decision-making’ has grown in disciplinary fields concerned with international development [6–8]. Goldman and Pabari [9] describe the development of policy and programming as taking place in stages, namely: agenda-setting, diagnosis, intervention selection, design or planning, implementation, assessment and continued learning. At various points in the policy cycle, evidence can be used to strengthen decision-making. Evidence can also be used to construct a theory of change, identify relevant outcomes, and define appropriate indicators of scale or quality in the design of an intervention, whether it is a policy or programme [9]. Additionally, evidence can be utilised as part of monitoring during implementation to evaluate progress, and essentially, for evaluating impact [9].

However, the intersection of disability-inclusion and evidence-based decision-making has seldom been examined and is not yet summarised outside of clinical settings. This is a notable shortcoming of the current research literature for three primary reasons. Firstly, what constitutes evidence – or even reliable evidence – in the context of different types of decision-making requires articulation. However, the concept of ‘evidence’ is not consistently

articulated in advocacy for evidence-based decision-making, with the implication being that western, empirical evidence may be privileged in people's working understandings of what constitutes evidence fit to inform decision-making. While this question is not the focus of the present paper, it is an important one because non-scientific, or scientific but not empirical, forms of evidence that are produced by the active involvement and consultation of people with disabilities by governments, as recommended in the United Nations Convention on the Rights of Persons with Disabilities (UNCRPD) [10], not only uphold this vulnerable population's rights to inclusion, but recognise them as "resource groups in society and contributors to development" [11, p. 4]. Yet whether such consultations are included within the concept of evidence-based decision-making is unclear.

Secondly, inattention to disability in policies and programmes in LMICs is often attributed to the lack of local evidence to inform such decision-making. Regardless of what is considered evidence, there is usually a shortage of it that is specific to people with disabilities and their needs and experiences in LMICs, partly because studies on the impact of interventions for people with disabilities are most commonly conducted in high-income countries (HICs) [3]. Moreover, the demographic data that is used to inform even the most fundamental planning by LMICs is often insufficient or irregular when it comes to disability, and disaggregation by disability status in larger datasets is difficult [12,13].

Thirdly, there is a general absence of formalised processes or documented best practices for drawing on evidence – even different types of evidence – to inform decision-making. This, coupled with the wide range of forces influencing the work of policy-makers and programme developers and managers, complicates efforts to strengthen the use of evidence in decision-making processes. Importantly, in LMICs, structural factors such as resource constraints, social norms, and post-colonial global power relations influence policy-making and programming landscapes in ways that may not fit as neatly with the evidence-

based decision-making models developed in HIC contexts [14,15]. Qualitative studies by researchers working in policy and implementation studies in LMICs [16–19] suggest that factors like trust, relationships, funding agendas and specific constellations of power, among other things, are prominent in shaping the development and implementation of policies and programmes. Furthermore, even when key stakeholders advocate for the use of evidence in the design and implementation of LMIC policies and strategies [5], there is a lack of guidance on how best to do this in relation to disability. Current approaches used by researchers, such as policy briefs, oral presentations, and reports, may be of limited impact, or their impact may be more significantly shaped by factors outside of the specific dissemination strategy, such as relationships, networks, and decision-maker interests. So, there is also a need for innovative approaches for communicating evidence to those who have the power to make decisions.

Each of the issues detailed above requires clarification. Some, such as what constitutes reliable evidence, may not be readily amenable to resolution because they relate to epistemology. Others though, such as how to improve *utilisation* of different types of evidence by key stakeholders, may be answerable through engagement with existing literature. Our work began with an even more basic question: does the current literature document how decision-makers in LMICs make decisions about disability in policy and programming, including the information and influences they value, and which processes are used? This scoping review aims to fill this knowledge gap by mapping and consolidating any literature which documents the sources of evidence, pathways, and processes involved in disability-inclusive decision-making in LMICs.

Materials and methods

Research questions

Primary research question(s)

The primary research question for this review was: "What is the nature of the literature concerning the use of evidence in governmental and non-governmental decision-making around disability-inclusion (i.e., what types of literature exist, where is this work published, what are its key concerns, and what kinds of key stakeholders are included)?"

Secondary research question(s)

This scoping review focused on examining existing evidence in the public domain with the intention of answering the following specific sub-questions:

1) What sources of information/evidence do key stakeholders draw on to inform their disability-relevant decisions in policy and programming?
2) Aside from information/evidence, what kinds of factors drive decision-making in disability-relevant policy and programming?
3) What are the barriers to and facilitators of evidence-based decision-making for LMIC governmental and non-governmental stakeholders in policy and programming in sectors including health, education, livelihoods, empowerment, and social inclusion?

Data sources and search strategy

An extensive review was undertaken to describe and analyse findings from a wide range of published literature, including both peer-reviewed and grey sources. This review was conducted according to a scoping review methodology, which involves five key steps, with the option of stakeholder consultations as a final step [20].

Firstly, we conducted thorough searches of various databases of peer-reviewed material, including CINAHL, ERIC, Scopus, Web of Science Social Sciences Citation Index,

MEDLINE(R), Embase Classic+Embase, PsycINFO, Cochrane, and CAB Global Health. Next, we conducted thorough online searches to gather relevant grey literature using the websites of the following large agencies and organisations: UNESCO, World Bank, ILO, WHO, UNICEF, SightSavers, CBM, International, Disability Alliance, UNHCR, Humanity and Inclusion, Inclusion International, Global Policy Forum, Save the Children, World Vision International, IRC and CRS, as well as supplementary Google Scholar searches. To improve the reporting and methodological quality of this review, we used the Preferred Reporting Items for Systematic Reviews and Meta-Analyses Extension for Scoping Reviews (PRISMA-ScR) (please see S1 File) and employed a comprehensive search strategy to mitigate for bias (please see S2 File). In the hand search of the grey literature, we selected relevant articles based on our inclusion and exclusion criteria and worked in one pair to screen the literature. Literature published before the year 1990 was not included in this scoping review. All searches were conducted between the 27th of August 2023 and the 14th of December 2023. The registered protocol for this scoping review is available at https://doi.org/10.17605/OSF.IO/D78HY.

The focus of this scoping review was evidence-based decision-making in respect of disability. As defined in the UNCRPD, a disability is a "long-term physical, mental, intellectual or sensory impairment, which, in interaction with various barriers, may hinder [a person's] full and effective participation in society on an equal basis with others" [21, p. 4]. The impairments in question include sensory, physical, intellectual, self-care, communication, and psychosocial impairments. As such, when piloting our search strategy, we recognised the need to engage with the literature on decision-making in mental health policy and programming, too. This was done to ensure that our scoping of the field was inclusive of a wide variety of impairments, including neurological and psychosocial impairments.

However, as is borne out in our results (see below) global public mental health has seen investment in, consideration of, and publishing about, policy-making which has not been mirrored in relation to the concerns of people with sensory, physical, intellectual, self-care, or communication impairments (for instance, in respect of rehabilitation, assistive technology, or early identification). We judged that if, in this analysis, disability was taken to be inclusive of psychosocial disability literature, it would have created a misrepresentation of the overall body of knowledge in the field.

As such, in our analysis and presentation of the results, we have made a distinction between the field of evidence-based decision making in disability and global public mental health broadly by grouping psychosocial disability in the mental health literature. Below, we have provided a summary of key features of all included studies (Part I), and then gone on to offer a deeper analysis of the studies related to disability but excluding psychosocial disability (Part II). This is not to imply that the authors consider psychosocial disability not to be a form of disability, but rather because:

1. It is not possible to meaningfully distinguish between literature focused on mental health *but not psychosocial disability*, and so all mental health literature was considered together;
2. The literature on mental health is far larger than the literature concerning people with sensory, physical, intellectual, self-care, or communication impairments; and
3. The concerns of people with sensory, physical, intellectual, self-care, or communication impairments may differ, in some systematic ways, from those documented in the global mental health literature, and so lessons learned from the latter literature may have limited applicability to the concerns of people with disabilities generally.

Study selection process

During study selection, the third step of the scoping review process, the authors used Rayyan.ai, a software platform specifically developed to facilitate collaborative evidence reviews in teams. Working in two pairs, we carefully assessed and selected literature based on our predetermined inclusion criteria, double screening all titles and abstracts. At the end of the full text article-screening phase, the reviewers reported a disagreement rate of 33%. These disagreements were resolved by the senior author.

Data extraction

For step four of this scoping review, all relevant information from each record was compiled in a spreadsheet. Information was extracted based on our research questions and included various details such as the authors' names and publication year, publication title, study design, types of literature, category of participants, level or ambit of decision-making, sectors and topics covered, impairment or health condition of focus, setting, data collection methods, sample size, aims, and a summarised study findings section. In addition, we prepared a more specific study findings section(s) that considered sources of evidence used, barriers and facilitators to using these sources of evidence, and other influences on disability-inclusive decision-making. Three reviewers assembled the data into a spreadsheet which was used to double-extract all articles, where the third reviewer (second author) extracted 100% of the articles. Any disagreements or uncertainties were resolved during team meetings, also involving the fourth (senior) author.

Data analysis

In the fifth and final step of this scoping review, we conducted data analysis that included numeric and qualitative content analyses. Qualitative content analysis was used to create codes and synthesise non-numerical data. Codes were created from inductively developed, condensed units of meaning using steps as described by Erlingsson and Brysiewicz [22]. Numeric analysis focused on quantitatively summarising five sections: the aims of papers, sources of evidence used in decision-making, influences on decision-making aside from evidence, as well as the barriers to and facilitators of using the aforementioned sources of evidence in decision-making. To ensure methodological rigour, analysis was completed by three of the authors, who developed analytical codes for the five sections mentioned above and documented emerging patterns. The final codes were validated by a fourth member of the research team, with additional discussions on codes held until consensus was reached.

Results

Results Part I: The Literature as a Whole, Inclusive of Mental Health

Summary of Findings

After duplicate removals, the search strategy produced a total of 3921 records. Following the careful review of titles and abstracts, we excluded papers that did not meet our inclusion criteria. Only the following types of records were included: grey literature reports; peer-reviewed studies; qualitative, quantitative, or mixed-method studies that reported primary research evidence exploring disability and decision-making in LMICs. Any papers that focused solely on HICs, were unrelated to disability, or did not concern high-level decision-making (e.g. respondents with disabilities describing access issues, limitations on

their participation, difficulties at home, income problems etc.) were excluded. As a result, 38 papers were found eligible for final analysis. We used the Preferred Reporting Items for Systematic Reviews and Meta-Analyses Extension for Scoping Reviews (PRISMA-ScR) guidelines to improve the quality of our methodology and reporting (Fig 1).

Fig 1. PRISMA-ScR.

Characteristics of records included in this review are summarised in Table 1. These records were arranged to display types of literature in two groups as follows: 1) the first 16 publications had a core focus on disability, and 2) the remaining 22 records had a core focus on mental health. For our in-depth analysis (Part II) we excluded articles that focused only on mental health policy-making and programming, as our key aim for this review was to explore how key stakeholders went about making decisions concerning disability-related policy or programming. For this scoping review, academics were not included as a population of interest. Once summary characteristics (Part I) have been described, only findings from records that related specifically to disability (other than psychosocial disability, as previously detailed) will be reported in the results section below.

Table 1. Summary of included literature.

Analysis category	First author & publication date	Type of literature	Stakeholder type (state, non-state, or both)	Sector(s) of participants	Impairment or health condition	Country or countries	Data collection methods	Sample size
Disability	Braun (2022)	Peer-reviewed	Both	Education	Childhood disability (general)	Tanzania	In-depth interviews (IDIs)	18
Disability	Brydges (2020)	Peer-reviewed	Both	Education; Health; Social Development; Other (Livelihoods)	Multiple disabilities	Uganda	Questionnaire; IDIs	25
Disability	Chibaya (2022)	Peer-reviewed	Both	Education; Health; Social Development; Other (Justice; Disability Affairs; Arts and Culture; advocacy organisations)	Multiple disabilities	Namibia	IDIs	14

Analysis category	First author & publication date	Type of literature	Stakeholder type (state, non-state, or both)	Sector(s) of participants	Impairment or health condition	Country or countries	Data collection methods	Sample size
Disability	Cleaver (2020)	Peer-reviewed	Both	Health; Social Development	Multiple disabilities	Zambia	IDIs	24
Disability	Grimes (2021)	Grey	Both	Education	Childhood disability (general)	Afghanistan, Bangladesh, Bhutan, India, Maldives, Nepal, Pakistan, Sri Lanka	Questionnaire; document review; IDIs	Not specified
Disability	Heidari (2021)	Peer-reviewed	Both	Health	Neurodevelopmental disability (Phenylketonuria)	Iran	IDIs; document review	38 (incl. unspecified number of academics)

Analysis category	First author & publication date	Type of literature	Stakeholder type (state, non-state, or both)	Sector(s) of participants	Impairment or health condition	Country or countries	Data collection methods	Sample size
Disability	International Disability Alliance (2017)	Grey	Non-state	Social Development; UN Agencies	Multiple disabilities	Ethiopia, Kenya, Nigeria, Togo, Bangladesh, India, Indonesia, Denmark, Italy, Sweden, Argentina, El Salvador, Peru	Questionnaire	Not specified
Disability	Jerwanska (2023)	Peer-reviewed	Both	Health; Social Development; Rehabilitation	Physical disabilities (rehabilitation)	Sierra Leone	Focus group discussions (FGDs)	37

Analysis category	First author & publication date	Type of literature	Stakeholder type (state, non-state, or both)	Sector(s) of participants	Impairment or health condition	Country or countries	Data collection methods	Sample size
Disability	Liechtenstein (2022)	Grey	Both	Other (humanitarian)	Multiple disabilities	Somalia	IDIs; document reviews; stakeholder analysis	9
Disability	Lyra (2023)	Peer-reviewed	Both	Health	Multiple disabilities	Brazil	Document review; IDIs	7
Disability	Morone (2014)	Peer-reviewed	State	Health	Sensory disability (visual impairment)	82 LMICs in Africa, America, Eastern Mediterranean, South East Asia, and the West Pacific	Questionnaire	Not specified

Analysis category	First author & publication date	Type of literature	Stakeholder type (state, non-state, or both)	Sector(s) of participants	Impairment or health condition	Country or countries	Data collection methods	Sample size
Disability	Najafi (2021)	Peer-reviewed	Both	Health, Education	Multiple disabilities	Iran	IDIs	21
Disability	Neill (2023)	Peer-reviewed	Both	Health	Multiple disabilities (rehabilitation)	47 LMICs and HICs across all WHO regions	IDIs; document review	65
Disability	Pillay (2022)	Peer-reviewed	State	Education; Health; Social Development	Neurodivergence (autism)	South Africa	IDIs	6
Disability	Shahabi (2020)	Peer-reviewed	State	Health	Multiple disabilities (rehabilitation)	Iran	IDIs; questionnaire	36
Disability	Wilbur (2021)	Peer-reviewed	Both	Education, Health, Social Development, Other (Federal Affairs; sexual and reproductive health)	Multiple disabilities (impaired self-care and activities of daily living)	Nepal	IDIs	12

Analysis category	First author & publication date	Type of literature	Stakeholder type (state, non-state, or both)	Sector(s) of participants	Impairment or health condition	Country or countries	Data collection methods	Sample size
Mental health	Araya (2012)	Peer-reviewed	Both	Health	Depression	Chile	IDIs	6
Mental health	Awenva (2009)	Peer-reviewed	Both	Health	Unspecified	Ghana	IDIs; FGDs	122
Mental health	Draper (2009)	Peer-reviewed	Both	Education; Social Development; Other (Justice)	Unspecified	South Africa	IDIs; questionnaire (WHO-AIMS)	64 for qualitative (n = unknown for quantitative)
Mental health	Hall (2020)	Peer-reviewed	Both	Social Development; Other (disability sector)	Unspecified	Timor-Leste	IDIs	85
Mental health	Hanlon (2017)	Peer-reviewed	State	Health	Unspecified	Ethiopia	IDIs	17

Analysis category	First author & publication date	Type of literature	Stakeholder type (state, non-state, or both)	Sector(s) of participants	Impairment or health condition	Country or countries	Data collection methods	Sample size
Mental health	Hann (2015)	Peer-reviewed	Both	Health	Unspecified	Sierra Leone	FGDs; IDIs	15 IDIs; 9 FGDs with up to 10 participants ea.
Mental health	Harpham (2005)	Peer-reviewed	Both	Health	Unspecified	Viet Nam	IDIs	4
Mental health	Hussain (2016)	Peer-reviewed	Both	Health	Unspecified	Pakistan	IDIs	15
Mental health	Iemmi (2022)	Peer-reviewed	Both	Health (Global mental health)	Unspecified	Twelve countries including 2 LMICs	Document review; IDIs	35
Mental health	Irmansyah (2020)	Peer-reviewed	Both	Health	Unspecified	Indonesia	IDIs	28

Analysis category	First author & publication date	Type of literature	Stakeholder type (state, non-state, or both)	Sector(s) of participants	Impairment or health condition	Country or countries	Data collection methods	Sample size
Mental health	Kleintjies (2013a)	Peer-reviewed	Both	Education; Health; Social Development; Other (Housing, Justice and Constitutional Development, Correctional Services)	Psychosocial disabilities	South Africa	IDIs	56
Mental health	Kleintjies (2013b)	Peer-reviewed	Non-state	Health	Psychosocial disabilities	Ghana, Kenya, Rwanda, South Africa, Tanzania, Uganda, Zambia	IDIs; document review	11
Mental health	Lempp (2018)	Peer-reviewed	Both	Health	Unspecified	Ethiopia, Nepal, Nigeria	IDIs	83

Analysis category	First author & publication date	Type of literature	Stakeholder type (state, non-state, or both)	Sector(s) of participants	Impairment or health condition	Country or countries	Data collection methods	Sample size
Mental health	Marais (2020)	Peer-reviewed	Both	Health	Unspecified	South Africa	IDIs; document review; observational data	7
Mental health	Marais (2021)	Peer-reviewed	Both	Health	Unspecified	South Africa	Observational data	10 sessions (n of participants not specified)
Mental health	Mirzoev (2012)	Peer-reviewed	Both	Health	Unspecified	Ghana, South Africa, Uganda, Zambia	IDIs	11

Analysis category	First author & publication date	Type of literature	Stakeholder type (state, non-state, or both)	Sector(s) of participants	Impairment or health condition	Country or countries	Data collection methods	Sample size
Mental health	Omar (2010)	Peer-reviewed	Both	Education, Health (mental health), Social Development, other (i.e., Correctional services, Justice, Housing)	Unspecified	Ghana, South Africa, Uganda, Zambia	IDIs; document review	247
Mental health	Samudre (2016)	Peer-reviewed	Both	Health	Unspecified	India	IDIs; FGD	27
Mental health	Stockwell (2005)	Peer-reviewed	Non-state	Education; Health; Other (academic)	Unspecified	Cambodia	IDIs	10
Mental health	Sulaberidze (2018)	Peer-reviewed	Both	Health	Unspecified	Georgia	IDIs; FGD; document review	41

Analysis category	First author & publication date	Type of literature	Stakeholder type (state, non-state, or both)	Sector(s) of participants	Impairment or health condition	Country or countries	Data collection methods	Sample size
Mental health	Votruba (2021)	Peer-reviewed	Both	Health; Other (academic)	Unspecified	South Africa	IDIs	21 (of which n=15 academics)
Mental health	Zwi (2011)	Peer-reviewed	Both	Health; Other (public media)	Unspecified	Solomon Islands	IDIs; document reviews	16

Types of literature

Of the 38 records that were included in our initial analysis, a total of 35 were published in peer-reviewed journals between 2005 and 2023. The remaining 3 records were grey literature reports published between 2017 and 2022. The combined total of LMIC research sites was over 100, with two publications covering more than 40 countries each. Of those studies in which countries were specified, the top study location was South Africa, representing 24% (n = 9) of papers reviewed, followed by four countries with the second highest number of mentions evenly split between Zambia, Uganda, and Ghana at 11% each (n = 4). Qualitative methods (n = 32) were used for the majority of included studies, while quantitative papers (n = 1) had the lowest frequency. The two most common types of data collection methods used were in-depth interviews at 89% (n = 34), and document reviews at 29% (n = 11), but many studies combined these methods. Focus group discussions were least commonly used, at 13% (n = 5).

Almost 60% of included papers were concerned with mental health topics (n = 22). The remaining 42% (n = 16) of records focused on disability more generally, often emphasising population groups such as women and children. Specific conditions or impairments were rarely cited but included depression (n =1), autism (n = 1), visual impairment (n = 1), and approaches to (predominantly physical) rehabilitation (n = 2). All (n = 22) of the papers focused on mental health programming or policy-making were peer-reviewed, while records which were specifically focused on disability included a mix of peer-reviewed (n = 13) and grey literature (n = 3). The sample size range for papers focused on mental health was between 6 and 65 participants, while the sample size ranged from 4 to 247 participants for the disability-focused literature.

Types of participants

Of the included records, 11% (n = 4) worked only with participants that were members of state (i.e., working within government). A similar number of papers (n = 3, 8%) of papers worked solely with non-state actors. Most records (n = 31, 81%) included a combination of both state and non-state actors. Many participating key stakeholders were involved in, or operated at, national levels of decision-making (n = 33, 87%), with only 5% (n = 2) based at a district level. For disability-related topics, more papers included only representatives from the government (n = 3) than in mental health-related papers (n = 1).

Types of sectors and topics covered

Overall, 84% (n = 32) of papers worked with participants from the health sector, although most studies included individuals from multiple sectors (including education, social development, housing, justice, economic and social affairs, and correctional services).

Results Part II: Disability-Focused Literature (Excluding Mental Health)

Summary of Findings

We identified 16 papers that focused on disability-related programming, policy-making, or both, and examined them in depth. From the 16 records, we identified 5 commonly-occurring aims in the literature. The most frequently occurring aim (n = 8, 50%) was the operationalisation of global approaches or commitments in LMIC settings, such as community-based rehabilitation (CBR) or the UNCRPD. The second most commonly explored topics were, equally, the implementation of policy, especially concerning barriers to implementation (n = 7), and investigations that sought to identify factors that contributed to conducive environments for disability policy-making and programming (n = 7). The third

most commonly observed objective was to explore the process of policy development (n = 6), followed by documentation of the role of collaboration among multiple key stakeholders (n = 4). Overall, the main aims of most of the included literature considered the topic of policy *implementation* over policy *formulation*.

The following sections describe the various types of evidence reportedly used in decision-making, and then goes on to detail the barriers to and facilitators of the use of each of these types of evidence. We also provide insight into other influences (besides formal evidence) on decision-making in disability policy and programming processes cited by study participants.

Cairney [23] describes evidence as a claim or argument supported by data and asserts that rigorous quantitative scientific research is sometimes understood as what constitutes evidence. However, Davies [24] expands this definition of evidence by providing seven additional classifications. In the analysis for this scoping review, we used Davies' [24] classification to categorise the sources of evidence reportedly used for decision-making processes in disability-related policies and programmes. Davies' [24] classifications include *statistical evidence*, *descriptive and experiential evidence*, *individual evaluations and research studies*, *implementation evidence*, *ethical evidence*, *economic and econometric*, and *research synthesis*.

Of the 16 papers we analysed in-depth, half (n = 8, 50%) of the analysed records documented the use of more than one type of evidence source [25–32]. In the following sections, each source and category of evidence identified in our analysis will be explored alongside its respective barriers and facilitators.

Aside from evidence, a range of other influences appeared to significantly shape decision-making in LMIC contexts (see Fig 2). We examined the 16 papers in this review for key influences on disability-related decision-making and identified 19 "other influences" in

total. From these 19 influences, 5 major themes emerged, namely: 1) government legislation, power dynamics and involvement (in decision-making), 2) stakeholder relationships, 3) local landscape, 4) funding, and 5) attitudes towards disability. These themes will be further explored in the subsequent sections to provide a comprehensive understanding of the factors influencing disability-related policy and programming decisions.

Fig 2. Disability-focused decision-making in LMIC contexts.

Sources of evidence and barriers and facilitators to use of evidence sources

Statistical evidence

The most common source of evidence reportedly used among key stakeholders was *statistical evidence.* This is described by Davies as evidence that is obtained from official statistics, administrative data, and surveys and which describes the nature, dynamics, and size of the issue in question [24]. Forty-four percent of papers (n = 7) showed that key stakeholders referenced population-level data such as statistics, survey, and census data as important sources of information for disability-related decision-making.

We analysed the literature gathered for review and observed various barriers to, and facilitators of, the use of statistical evidence in decision-making. Among the 17 barriers to the use of statistical evidence for decision-making, inaccurate or underestimated scientific data was most commonly cited. Jerwanska et al. [28, p. 1799], for instance, reported that key stakeholders with influence on decision-making would “not accept the prevalence data on disability according to the national census conducted” because they considered the information to be inaccurate. Similarly, Liechtenstein [29] recounted the criticisms of key stakeholders who learned that reliable census or disaggregated data to guide resource

distribution and policy-making, in work concerning children with disabilities, was lacking overall.

Other commonly cited barriers to the use of statistical evidence included the following, equally: inconsistent or outdated definitions of disability regularly being used in census tools; the lack or inappropriateness of locally-relevant research; and insufficient scientific knowledge, experience, and expertise among key stakeholders. Additionally, although cited only once, a notable barrier was the costliness of data collection tools, specifically in terms of expanding the number of census questions to adequately identify and quantify disability prevalence [33].

On the other hand, four facilitators of the use of statical evidence in decision-making were identified in the literature. These were: financial resources; properly capacitated data capturers; electronic information management systems strengthening; and standardised methods for collecting and analysing disability-related data. For example, Wilbur et al. reported on the barriers to menstrual hygiene management (MHM) faced by people with disabilities and reflected on the fact that key stakeholders felt that "investment is required to generate evidence" [32, p. 2]. This highlights the role that resources, such as finances, play in the collection of statistical evidence. The capacitation of data capturers was also cited as important for ensuring that the quality of data collected by censuses was adequate. This improved data would, in turn, encourage trust in and the increased use of this kind of evidence among key decision-making stakeholders [28].

Additionally, stakeholders noted that the strengthening of electronic information management systems could facilitate the increased trust in and use of statistical evidence in some countries, because stronger information systems would support "ongoing improvements in identifying disability, data collection, monitoring and quality assurance" [25, p. 6]. Finally, stakeholders reported that the accuracy and reliability of evidence could be improved by

developing and disseminating standardised methods for collecting and analysing disability-related data. This was detailed by Grimes et al., who further asserted that if the Washington Group on Disability Statistics (WG) questions were used across all data collection efforts, the "generation of consistent and comparable data" would be facilitated, supporting its use as evidence in policy and programming [25, p. 6].

Descriptive and experiential evidence

The second most commonly cited source of evidence aligns with Davies' category of *descriptive and experiential evidence*. This classification is defined by Davies [24] as intuitive or "tacit" knowledge and the lived experiences that are used to shed light on the nature, dynamics, and scale of an issue. Within the disability-focused literature we analysed, this type of knowledge was characterised by the theme *disability representation*. This theme included: 1) *lived experiences,* and 2) *formally recognised disability advisors*.

Under the theme disability representation, *lived experiences* was cited as a source of evidence in a limited number of reviewed records. We defined this category as the experiences of people with disabilities, and those of their families and caregivers, who are self-representative and do not formally constitute associations or organisations but are able to assist in gathering research evidence and hold tacit knowledge that may be useful to key stakeholders in high-level decision-making. An example of this form of evidence was documented by Grimes et al. and described as "the practice of consulting and involving children with disabilities, their families and caregivers in decision-making processes" [25, p. 7]. The authors found that the incorporation of this type of evidence was limited at local and national levels of policymaking and programming, but was nonetheless perceived as relevant and necessary in efforts toward the development of inclusive education.

We created the coding category of *formally recognised disability advisors* to refer to disability representative bodies, such as organisations of persons with disabilities (OPDs) (also known as disabled people's organisations or DPOs), disability focal point persons (FPPs), expert disability advisors, and the medical associations and national committees that collaborate with policy-makers on disability-related matters. This source of evidence was cited only in a minority of records. The representative bodies described by participants in this category were either formalised structures within governing bodies or independent, non-governmental bodies (NGOs). Formally-recognised disability advisors fulfilled tasks such as consultations with government departments, policy formulation, tool development, primary or secondary scientific data collection, advocacy, or report-writing [29–32,34,35].

When it came to disability representation in decision-making, the most commonly cited barriers were the low social and political prioritisation and profile of disability, and the lack of consultation or inclusion of relevant stakeholders, particularly people with lived experiences [32]. Jerwanska et al. [28] specifically noted the exclusion of people with disabilities from dialogues around policy-making for disability. This exclusion seemed to counteract the efforts of disability representative bodies in terms of being a useful source of evidence.

Insufficient scientific knowledge and skills among members of formally recognised disability advisory bodies, and weak collaborative capacity between government sectors and these advisory bodies commonly led to their inability to influence decision-making. Moreover, the poor structure and capacity of disability advisory bodies in terms of policy-related technical skills appeared to result in less influence in decision-making spaces. As an example, Cleaver et al. reported that FPPs did not have sufficient power because they were "ministry employees in low level positions who were unable to access ministry information or influence decisions" [34, p. 5], owing to a lack of detail provided in a national strategic

plan regarding their function. Additionally, insufficient funding and the lack of shared identity and coalition among people with disabilities was seen to reduce their influence on policy, as it inhibited their ability to engage politically influential actors [31].

We identified 14 facilitators of the use of *disability representation* as evidence in policy-making and programme development. Most commonly highlighted was the importance of collaborative relationships between national government and non-governmental stakeholders. The next most frequently noted facilitator was key stakeholders' increased awareness of and capacity for managing disability inclusion. The availability of financial resources to sustain the functionality of these advisory bodies, improved disability-inclusion in wider policies, and political will were seen as equally important enablers of the incorporation of formally recognised disability advisors as a source of evidence.

Individual evaluations and research studies

An additional source of evidence observed in this review was desk research, described by Davies [24] as *individual evaluations and research studies*. Under this code, we included papers (n = 3, 19%) that cited the use of secondary data and called this theme *desktop research*. For example, Heidari et al. [26] reported that research from other countries was used to inform the adaptation and development of international standards for a novel phenylketonuria (PKU) screening programme in Iran.

Barriers that participants cited against the use of evaluation and research study evidence included scientific data that was inconsistent and unreliable, and the invisibility/limited accessibility of existing resources, particularly published data. However, the availability of scientific human resources (such as academic and medical personnel), the increased awareness and capacity of key stakeholders in disability-related research matters, and the establishment of collaborative relationships between researchers and key decision-

making stakeholders were all cited as factors that facilitated this use of evidence. Moreover, pressure from relevant stakeholders (like OPDs and people with disabilities working with government and civil society organisations (CSOs) and NGOs external to government) and the consistent reporting of methodologically-sound, disability-disaggregated data were also evenly split as cited facilitators of the use of this kind of evidence.

Implementation evidence

The fifth most common source of evidence reported in reviewed records was classified as *implementation evidence,* which is described by Davies [24] as documentation of the ways in which obstacles to the adoption of comparable policies have been overcome and such policies have been successfully implemented. This category was slightly adapted in the context of our review to include international guidelines (n = 2, 13%). Examples of sources reportedly used by key stakeholders were World Health Organisation (WHO) guidelines, specifically those focused on rehabilitation and Universal Health Coverage (UHC), and WHO checklists for (health and other) system assessments [31,36].

Barriers to the use of implementation evidence include influential key stakeholders' refusal to adopt international recommendations if they considered these irrelevant to their local context. Brydges et al. [37] also noted that participants had commented on the insuffic13ient human resources and funding available in LMIC settings, meaning that adherence to international guidelines can result in a dependence on volunteers who may later leave. Additionally, the insufficient transfer of technical knowledge from international to national and sub-national actors was observed as another hindrance to implementation in LMICs. Within a sample of influential key stakeholders who stated that they knew about the CBR approach, for example, "70% said that they were unfamiliar with the CBR *guidelines,* which provide concrete guidance on how to implement a CBR [programme]"[37, p. 1607].

The limited sustainability of programming influenced by international guidelines or principles due to changing political circumstances, such as the withdrawal of international support because of shifts in donor priorities, for example, also affects the likelihood that LMIC-based decision-makers will adopt international guidelines as a source of evidence [21, 29]. These findings suggest a notable trend toward a preference for using local population-level data as evidence in the process of high-level decision-making.

Conversely, strong collaborative relationships between multisectoral national and sub-national actors, and the easy availability of international guidance – such as WHO guidelines as a rationale for promoting rehabilitation's integration into the health system, for instance [31] – could facilitate the incorporation of implementation evidence into decision-making. Additional facilitators in this area included the active involvement of important stakeholders, such as government leaders who champion disability-related matters in political arenas, good governance, and the availability of standardised tools. A summary of the aforementioned barriers and facilitators to the use of specific forms of evidence is illustrated below (Fig 3 and Fig 4).

Fig 3. Most frequently cited barriers to sources of evidence used in decision-making.

Fig 4. Most frequently cited facilitators to sources of evidence used in decision-making.

Other influences on decision-making

Next, we detail each "other influence" on decision-making reported in the analysed literature (Fig 5) in turn, providing examples from the literature.

Fig 5. Hierarchy chart representing other influences on decision-making.

Government legislation, power dynamics and involvement

The first “other influence” on decision-making was themed “government legislation, power dynamics and involvement” consisted of categories such as legal frameworks, government involvement, policy windows, power dynamics due to hierarchical political structures, and national prioritisation (or de-prioritisation) of disability-related matters.

In terms of the influence of *legal frameworks*, Pillay et al. [38] and Wilbur et al. [32] noted that a national constitution can be used to mandate policy development in support of people with disabilities and uphold their rights as citizens. Wilbur et al. [32] argued that this was rooted in a state’s desire to maintain the identity of a country committed to equality.

Policy windows – where an unexpected opportunity occurs in the process of policy development that allows advocates to draw attention to their issues – had a notable influence on disability-related policy and programming within included studies, because these windows generated opportunities for shaping decision-making. Policy windows were reportedly especially significant for awareness-raising and advocacy [31]. For example, Heidari et al. [26] described the important role of an individual who was able to convince policy-makers to create a policy window, which later led to the development and launch of a successful PKU screening programme in Iran. Conversely, the inability to motivate for prioritisation in agenda-setting negatively affected the progression of disability-related initiatives [31].

Closely related to the idea of “policy windows” are the complex *power dynamics* inherent in many governments due to deeply hierarchical political structures and *national prioritisation*. Though these categories are distinct, there was a notable overlap of these themes in the records reviewed. This is likely due to the perceived interplay between the roles of key stakeholders with decision-making power, their attitudes towards disability initiatives, and the actions (or a lack thereof) that they take. One study specifically cited the amplified

roles of policy-making actors, particularly at the national level [35]. Similarly, another study [39] reported that national governing bodies carried immense influence, and unless government was interested in and took responsibility for disability inclusion, disability-related programmes did not receive sufficient support. This lack of support, especially in financial terms, negatively affected the sustainability of programmes for disability inclusion.

With regard to the influence that *government involvement* had on decision-making, this was reportedly either very helpful for or a hinderance to the development of disability policies or initiatives. Liechtenstein stated that “to really have a successful disability inclusion project, it has to be owned by the system, so by the government, by the local authorities” because the government “has the power to either jeopardise a project’s success”, or to help it along [29, p. 11]. In a different study, the national Ministry of Health and Sanitation (with the support of an international organisation) held the most significant power in shaping strategies and policies [30]. Similarly, shifting the responsibility for health and rehabilitation services from a different ministry – the Ministry of Social Welfare, Gender and Children’s Affairs (MSWGCA) – to the national Ministry of Health and Sanitation yielded improvements for people with disabilities [28].

Stakeholder relationships

Stakeholder relationships were another influence on decision-making. This theme consisted of categories including the *involvement of relevant non-governmental stakeholders*, *strategic alliances*, and *collaborative capacity*. *Strategic alliances* reportedly happened between non-governmental stakeholders, organisations, and agencies. In a study concerning the promotion of eye health in LMIC settings, it was reported that since “local stakeholders are key for securing policy support, specific actions are needed to promote their participation” [30, p. 195], alongside the encouragement of local community stakeholder and

local NGO engagement through capacity-building initiatives. These alliances took the form of partnerships between local business communities and international development agencies, as well as collaborations between OPDs, policy-makers, and implementers [40].

Morone et al. [30] noted the significant role that global stakeholders, such as foreign investors and international donors, played in international cooperation, particularly in terms of exerting “positive” (i.e., toward more disability inclusion) pressure on policy-makers. According to Morone et al. [30], the *involvement of relevant stakeholders* such as the WHO, volunteer organisations, and foundations were impactful on decision-making for national policies for blindness prevention in low-income countries (LICs). However, this did not necessarily translate for LMICs and upper-middle income countries (UMIC), where the private sector played a more influential role in this regard [30] .

The presence or absence of *collaborative capacity* also influenced decision-making. Neill et al. [31] noted that the capacity to influence decision-makers is strongest when there is a shared goal [or] agenda, highlighting the importance of collaborative abilities and alignment between different groups. In the process of mapping potential stakeholders in physical rehabilitation financing in Iran, Shahabi et al. [39] noted that although some entities could potentially be influential in decision-making spaces (such as the WHO, national welfare organisations, and NGOs), these groups had few opportunities to work together and were therefore less influential in decision-making.

Local landscape

The theme “local landscape” considers the sociocultural, political, and economic events and contexts that had an influence on decision-making, according to participants in included studies. Examples of these were “post-conflict political influences” (e.g. dependence on international aid organisations) [37], massive disease outbreaks that happened in previous

years [26], international guidelines and global trends that improved the process of implementation for disability inclusion [29], and the political context in general. An example of a political contextual factor was the ratification of the UNCRPD by Brazil in 2008, which Lyra et al. [35] noted had provided an important stimulus for the uptake of disability-inclusive policies. In addition, a strong disability-related social movement played a significant role in bringing about the 2002 adoption of the national health policy by advocating for inclusion and the right to health. However, once political circumstances changed and a fiscally and socially conservative governing party came into power, policies and programming relevant to disability lagged, further demonstrating the importance of the political context on progress toward inclusion.

Funding

The theme "funding" considers the availability of financial resources in support of disability policy or programming. In the context of inclusive education reforms, for example, Braun [36] noted the considerable expense that the establishment and continuation of teacher training programmes carried, which required substantial financial investment in infrastructure and human capital but more importantly, political commitment. Jerwanska et al. [28] noted the negative impact that insufficient government funding had had on health service delivery. Brydges et al. [37] and Neill et al. [31] similarly referenced the unsustainable dependence on international funding for rehabilitation services that were based on WHO guidelines but were being implemented in low-resource settings.

Attitudes towards disability

This theme concerns stigma, discrimination, and negative overall attitudes and beliefs towards disability, and how these have influenced decision-making. Grimes et al. [25] noted

that the discrimination and stigma held against children with disabilities could influence not only learning environments and services but just as importantly, policy and programming. Lastly, Braun [36] noted that policy actors reported that individuals within and beyond the education system often carry unhelpful and uninformed attitudes, such as the belief that students with disabilities should be kept in the home instead of being allowed to receive an education, which decrease the chances of disability-inclusive policy-making and programming.

Discussion

This scoping review provides a novel evaluation of literature concerning the use of evidence in governmental and non-governmental decision-making processes around disability-inclusion in LMICs. Our findings highlight a significant gap: the insufficient availability of research focused specifically on the *formulation and development* of disability-related policies and programmes. This was observed in only 16 records (3 of which were grey literature) retrieved from an extensive search. Notwithstanding its relatively small size, this body of work yields important insights, notably around the definition and use of evidence in decision-making, the factors outside of evidence that impact decisions, and the key stakeholders involved in and processes surrounding the development of disability-related initiatives. The main insights pertain to those sectors most represented in the literature, the documentation of policy and programme development processes, and the main sources of evidence used in and other influences on decision-making toward disability inclusion.

Firstly, this review demonstrates that the health sector is most widely represented in the literature on decision-making in relation to disability in LMICs. This is perhaps unsurprising, given the focus of much disability policy and programming in LMICs on

rehabilitation [41–43]. However, this finding also suggests that disability as a topic is still medicalised, resulting in poorer visibility in other sectors like social protection and education [44]. By extension, as a population people with disabilities appear to still be viewed from a medicalised perspective. Many public health interventions depend for their success on the intersectoral participation of sectors including labour, welfare and social assistance, housing, transportation, and education [45,46]. Considering the fact that disability affects individuals in diverse and varied ways, but in all aspects of their lives, it is important that intersectoral collaboration is an early and central aspect of any disability-related programme or policy. The evaluation of disability-inclusive policies and programmes could be a promising site for promoting intersectoral collaboration because evaluative processes provide numerous opportunities to bring together stakeholders with specific roles in the implementation of a policy or programme, and whose interests are impacted by it, both during the planning stages and during the evaluative process itself. Evaluations can be planned as deliberate procedures in which stakeholders from many sectors consider and balance differing viewpoints, agree on the main subject of the assessment, and decide which outcomes to measure [45]. This approach would be beneficial for the development of disability policy and programming in LMICs.

Secondly, the literature identified in this review explores policy and programme implementation, or the lack thereof, as opposed to policy *formulation*. An optimistic interpretation of this finding is that it suggests that many LMIC countries have existing disability policies to be reflected upon. However, the failure to document policy and programme development processes means that the presence or absence of different types of evidence – and the process of how decisions are made – remains opaque. To foster better knowledge and more informed decision-making among those in charge of government operations, evaluation experts are tasked with providing retrospective evaluations of the

execution, output, and result of government actions [47]. Evaluation entails retrospective analysis in order to steer ahead more effectively. This process assists with separating what is valuable from what is not. Additionally, because evidence develops over time, we need to understand and identify potential evidence “entry points” during policy and programme reviews and updates, in order to improve practices in alignment with emerging knowledge. According to Vedung [47], all forms of assessment are predicated on the notion that views, beliefs, intents and judgements play a significant role in political and administrative activity. It is worth considering and understanding how these factors work, but this can only be achieved through thorough documentation of the policy and programme development processes.

Thirdly, government legislation, dynamics, and interest in disability inclusion were reported as the most powerful influences on the development of progressive disability-related policy and programming. Gilson and colleagues [48] acknowledge that governance is a multi-layered, intricate process that affects policy enactment, decision-making, and service delivery in the field of health systems research. Thus, “whole-systems” awareness requires knowledge and comprehension of both the “software” (e.g., values, norms, and relationships) and “hardware” (e.g., finances, resources, and infrastructure) aspects of a system [48]. In our review, the national legislation of a country and actions resulting from the involvement of government could make or break programmes in terms of their progress and sustainability. Disability initiatives grew or flourished when the state took responsibility for them and demonstrated good leadership.

Local statistical evidence from censuses and survey data gathered also reportedly played a role in the development of disability policies and initiatives. A preference for local data combined with the lack of uniformity or standardisation of data collection tools nationwide may explain the regular use of WHO guidelines and tools by some influential key

stakeholders, and the appraisal of such to be ill-fitting for their local context, by others. Cairney [44] asserts that the framework for policy formation is established beliefs, therefore, any new evidence regarding the efficacy of a policy must be accompanied by a change in emphasis and persuasive arguments. At times, social or economic crises may serve as catalysts to transfer focus from one matter to another, aided by specific forms of evidence. However, substantial policy transformations are said to be uncommon [44].

Where collaboration and strong relationships among relevant stakeholders were reported, particularly between local and international non-governmental bodies, the cumulative influence of pressure from these entities was sometimes enough to sway government decisions. According to literature on policy-making, but with applicability to programming too [44], these kinds of decisions often occur in unpredictable and complex environments. These environments are characterised by the following features: a wide variety of organisations and individuals influencing policy at different levels of government; a multitude of rules and norms observed across different contexts; close connections (or "networks") between policy-makers and influential actors; the prevalence of particular beliefs or "paradigms" in discussions; and situations or events that have the potential to swiftly capture the attention of policy-makers. It seems possible that collaborative efforts among local and international NGOs who have access to and make use of higher quality disability data may have the power to significantly influence government decisions in complex multi-level policy environments.

Based on our scoping review findings, we recommend the encouragement of multidisciplinary approaches that integrate the perspectives of varied sectors including (but not limited to) health, education, social sciences, construction, and labour in the work of disability policy and programming. Governments should look to consistently involve a diverse range of stakeholders, including people with disabilities and advocacy groups, in the

early stages of policy and programme formulation to ensure effective inclusivity. Standardising definitions of disability and data collection tools across LMIC research while also adapting international guidelines to local contexts may help to address inconsistencies in scientific research and data collection tools. Clear, country-specific implementation strategies, robust monitoring and evaluation frameworks, and indicators disaggregated by age, gender, and disability status are essential for creating useful statistical evidence and encouraging the use of this data in efforts towards the development of disability policy or programming in LMICs. Moreover, strengthening leadership, with a focus on both the "software" and "hardware" aspects of governance, is crucial for sustainable progress in disability initiatives.

Limitations

In the process of conducting this scoping review, we relied on the data obtained and extracted from the literature that met our inclusion criteria. However, we acknowledge that by basing this review solely on papers published in English, we may have lost valuable insights, especially those emerging from Latin or Francophone countries. Finally, due to time constraints and the desire to present succinct findings, we documented only the most frequently occurring and salient barriers to, and facilitators of, evidence use, and other influences on decision-making, and limited these to the top five most commonly occurring themes.

Conclusion

The findings from this scoping review shed light on documented influences affecting the decision-making process, types of evidence that inform disability-related initiatives in

LMICs, and existing knowledge about what may support or hinder these decision-making processes. These findings, while limited, nonetheless provide clear avenues for supporting disability-inclusive policy and programming by highlighting key barriers to the utilisation of evidence, which can be targeted with interventions. This review also highlights factors which could facilitate increased consideration of disability in decision-making, which could be amplified through targeted work with decision-makers. Finally, this work shows how a multitude of information types are considered under the umbrella of "evidence" by decision-makers and describes structural drivers of decision-making which may act outside of or as a complement or competition to the use of (empirical) evidence to inform decision-making. The powerful influence of government legislation, power dynamics, and (level of) interest in disability inclusion, combined with a reported lack of faith in local evidence, suggests that stakeholders may make specific decisions due to insufficient access to, or trust in, the available evidence. This possibility should be explored further in future research. Stakeholders seeking to support practices around evidence-based decision-making in LMICs must be cognisant of these differing framings of the concept of evidence, and the importance of influences outside of evidence on decision-making behaviours, in the development of relevant interventions.

Supporting information

S1 File. Preferred reporting items for systematic reviews and meta-analyses extension for Scoping Reviews (PRISMA-ScR) checklist.

S2 File. Search strategy.

Acknowledgements

We would like to thank Vittoria Lutje of the Liverpool School of Hygiene and Tropical Medicine for her support in conducting systematic database searches.

References

1. WHO. Global report on health equity for persons with disabilities. 2022.
2. Rivas C, Tomomatsu I, Gough D. The many faces of disability in evidence for policy and practice: Embracing complexity. Vol. 17, Evidence and Policy. Policy Press; 2021. p. 191–208.
3. Saran A, White H, Kuper H. Evidence and gap map of studies assessing the effectiveness of interventions for people with disabilities in low-and middle-income countries. Vol. 16, Campbell Systematic Reviews. Wiley Blackwell; 2020.
4. White H. Improving social inclusion and empowerment for people with disabilities in low-and middle-income countries: why does it matter and what works? Why does social exclusion and lack of empowerment matter for people with disabilities? 2012.
5. Swartz L. Science for social justice: Thinking more broadly about inclusion. S Afr J Sci [Internet]. 2023 Jan 31 [cited 2024 May 20];119(1/2). Available from: https://sajs.co.za/article/view/15447
6. Baba V V., HakemZadeh F. Toward a theory of evidence based decision making. Management Decision. 2012 May;50(5):832–67.

7. Behague D, Tawiah C, Rosato M, Some T, Morrison J. Evidence-based policy-making: The implications of globally-applicable research for context-specific problem-solving in developing countries. Soc Sci Med. 2009;69(10):1539–46.

8. Umbach G, Guidi CF, Russo M. Brief policy Evidence-based policy-making: From data to decision-making executive training seminar global governance programme & school of transnational governance. 2018.

9. Goldman I, Pabari M. An introduction to evidence-informed policy and practice in Africa. In: Using Evidence in Policy and Practice [Internet]. London: Routledge; 2020 [cited 2024 May 20]. p. 13–33. Available from: https://www.taylorfrancis.com/books/9781003007043/chapters/10.4324/9781003007043-2

10. International Disability Alliance. Participation of Organisations of Persons with Disabilities in Development Programmes and Policies [Internet]. 2019 [cited 2024 May 27]. Available from: https://www.ohchr.org/sites/default/files/Documents/Issues/IntOrder/GlobalGovernanceSpaces/InternationalDisabilityAlliance.pdf

11. Division for Social Policy Development (DSPD), Department of Economic and Social Affairs (DESA). Toolkit on Disability for Africa: Disability-Inclusive Development [Internet]. 2017 [cited 2024 May 24]. Available from: https://www.un.org/esa/socdev/documents/disability/Toolkit/Disability-inclusive-development.pdf

12. Banks LM, Kuper H, Polack S. Poverty and disability in low-And middleincome countries: A systematic review. Vol. 12, PLoS ONE. Public Library of Science; 2017.

13. Mitra S, Chen W, Hervé J, Pirozzi S, Yap J. Invisible or Mainstream? Disability in Surveys and Censuses in Low-and Middle-Income Countries. 2021.

14. Powers T. On power and policy in post-colonial Africa: an introduction. Vol. 40, Journal of Contemporary African Studies. Routledge; 2022. p. 317–32.

15. Health Organization Regional Office for Europe W. Making decisions on public health: a review of eight countries. 2004.

16. Hoag C, Hull M. A Review of the Anthropological Literature on the Civil Service [Internet]. 2017. Available from: http://econ.worldbank.org.

17. Hodder R. Global South and North: Why Informality Matters. New Global Studies. 2016;10(2):113–31.

18. Hodder R. Emotional Bureaucracy. New York: Routledge; 2017.

19. Hull MS. Government of Paper. The Materiality of Bureaucracy in Urban Pakistan [Internet]. Vol. 37, South Asia: Journal of South Asian Studies. 2014. 361–362 p. Available from: http://www.tandfonline.com/doi/abs/10.1080/00856401.2014.909348

20. Mak S, Thomas A. An Introduction to Scoping Reviews. J Grad Med Educ. 2022 Oct 1;14(5):561–4.

21. United Nations Convention on the Rights of Persons with Disabilities. Convention on the Rights of Persons with Disabilities and Optional Protocol UNITED NATIONS. 2006.

22. Erlingsson C, Brysiewicz P. A hands-on guide to doing content analysis. Vol. 7, African Journal of Emergency Medicine. African Federation for Emergency Medicine; 2017. p. 93–9.

23. Cairney P. The Politics of Evidence-Based Policy Making. 1st ed. Palgrave Pivot; 2016.

24. Davies P. Evidence-Based Policy-Making (EBPM): Enhancing the use of evidence and knowledge in policy making. PSPPD Policy Brief 1 [Internet]. 2011 Jul [cited 2024 May 20]. Available from: https://africacentreforevidence.org/wp-content/uploads/2019/02/PSPPD-2011-Evidence-Based-Policymaking-EBPM-enhancing-the-use-of-evidence-and-knowledge-in-policy-making.pdf

25. Grimes P, dela Cruz. A. Mapping of Disability-Inclusive Education Practices in South Asia [Internet]. Kathmandu; 2021. Available from: www.unicef.org/rosa/

26. Heidari A, Arab M, Damari B. A policy analysis of the national phenylketonuria screening program in Iran. BMC Health Serv Res. 2021 Dec 1;21(1).

27. International Disability Alliance. The Case Study on the Engagement of Organizations of Persons with Disabilities (DPOs) in Voluntary National Reviews [Internet]. 2017 [cited 2024 May 20]. Available from: https://www.internationaldisabilityalliance.org/sites/default/files/global_report_on_the_participation_of_organisations_of_persons_with_disabilities_dpos_in_vnr_processes.docx

28. Jerwanska V, Kebbie I, Magnusson L. Coordination of health and rehabilitation services for person with disabilities in Sierra Leone–a stakeholders' perspective. Disabil Rehabil. 2023;45(11):1796–804.

29. Liechtenstein C. Still left behind? Growing up as a child with a disability in Somalia Disability inclusion: stakeholder, perception, and implementation of in Save the Children's projects in Somalia.

30. Morone P, Camacho Cuena E, Kocur I, Banatvala N. Securing support for eye health policy in low- and middle-income countries: Identifying stakeholders through a multi-level analysis. Vol. 35, Journal of Public Health Policy. Palgrave Macmillan Ltd.; 2014. p. 185–203.

31. Neill R, Shawar YR, Ashraf L, Das P, Champagne SN, Kautsar H, et al. Prioritizing rehabilitation in low- and middle-income country national health systems: a qualitative thematic synthesis and development of a policy framework. Int J Equity Health. 2023 Dec 1;22(1).

32. Wilbur J, Scherer N, Mactaggart I, Shrestha G, Mahon T, Torondel B, et al. Are Nepal's water, sanitation and hygiene and menstrual hygiene policies and supporting documents inclusive of disability? A policy analysis. Int J Equity Health. 2021 Dec 1;20(1).

33. Najafi Z, Abdi K, Khanjani MS, Dalvand H, Amiri M. Convention on the rights of persons with disabilities: Qualitative exploration of barriers to the implementation of articles 25 (health) and 26 (rehabilitation) in Iran. Med J Islam Repub Iran. 2021;35(1):1–9.

34. Cleaver S, Hunt M, Bond V, Lencucha R. Disability Focal Point Persons and Policy Implementation Across Sectors: A Qualitative Examination of Stakeholder Perspectives in Zambia. Front Public Health. 2020 Sep 15;8.

35. Lyra TM, Veloso De Albuquerque MS, Santos De Oliveira R, Morais Duarte Miranda G, Andra De Oliveira M, Eduarda Carvalho M, et al. The National Health Policy for people with disabilities in Brazil: an analysis of the content, context and the performance of social actors. Health Policy Plan. 2022 Nov 1;37(9):1086–97.

36. Braun AMB. Barriers to inclusive education in Tanzania's policy environment: national policy actors' perspectives. Compare. 2022;52(1):110–28.

37. Brydges C, Munro LT. The policy transfer of community-based rehabilitation in Gulu, Uganda. Disabil Soc. 2020 Oct 21;35(10):1596–617.

38. Pillay S, Duncan M, de Vries PJ. 'We are doing damage control': Government stakeholder perspectives of educational and other services for children with autism spectrum disorder in South Africa. Autism. 2024 Jan 1;28(1):73–83.

39. Shahabi S, Ahmadi Teymourlouy A, Shabaninejad H, Kamali M, Lankarani KB. Financing of physical rehabilitation services in Iran: A stakeholder and social network analysis. BMC Health Serv Res. 2020 Jul 1;20(1).

40. Chibaya G, Naidoo D, Govender P. Exploring the implementation of the United Nations Convention on the Rights of Persons with Disabilities (UNCRPD) in Namibia. Perspectives of policymakers and implementers. South African Journal of Occupational Therapy. 2022;52(1).

41. Neill R, Shawar YR, Ashraf L, Das P, Champagne SN, Kautsar H, et al. Prioritizing rehabilitation in low- and middle-income country national health systems: a qualitative thematic synthesis and development of a policy framework. Int J Equity Health. 2023 Dec 1;22(1).

42. Bright T, Wallace S, Kuper H. A systematic review of access to rehabilitation for people with disabilities in low-and middle-income countries. Vol. 15, International Journal of Environmental Research and Public Health. MDPI AG; 2018.

43. McPherson A, Durham J, Richards N, Gouda H, Rampatige R, Whittaker M. Strengthening health information systems for disability-related rehabilitation in LMICs. Health Policy Plan. 2017;32(3):384–94.

44. Cairney P. The Politics of Evidence-Based Policy Making [Internet]. Vol. 1. Oxford University Press; 2017. Available from: http://oxfordre.com/politics/view/10.1093/acrefore/9780190228637.001.0001/acrefore-9780190228637-e-268

45. Kriegner S, Ottersen T, Røttingen JA, Gopinathan U. Promoting intersectoral collaboration through the evaluations of public health interventions: Insights from key informants in 6 European countries. Int J Health Policy Manag. 2021 Feb 1;10(2):67–76.

46. World Health Organization. Early Childhood Development and Disability: A discussion paper. 2012.

47. Vedung E. Public policy and program evaluation. Transaction; 2000.

48. Gilson L, Barasa E, Nxumalo N, Cleary S, Goudge J, Molyneux S, et al. Everyday resilience in district health systems: Emerging insights from the front lines in Kenya and South Africa. BMJ Glob Health. 2017;2(2).

Figure 1

Click here to access/download;Figure;Fig 1.tif

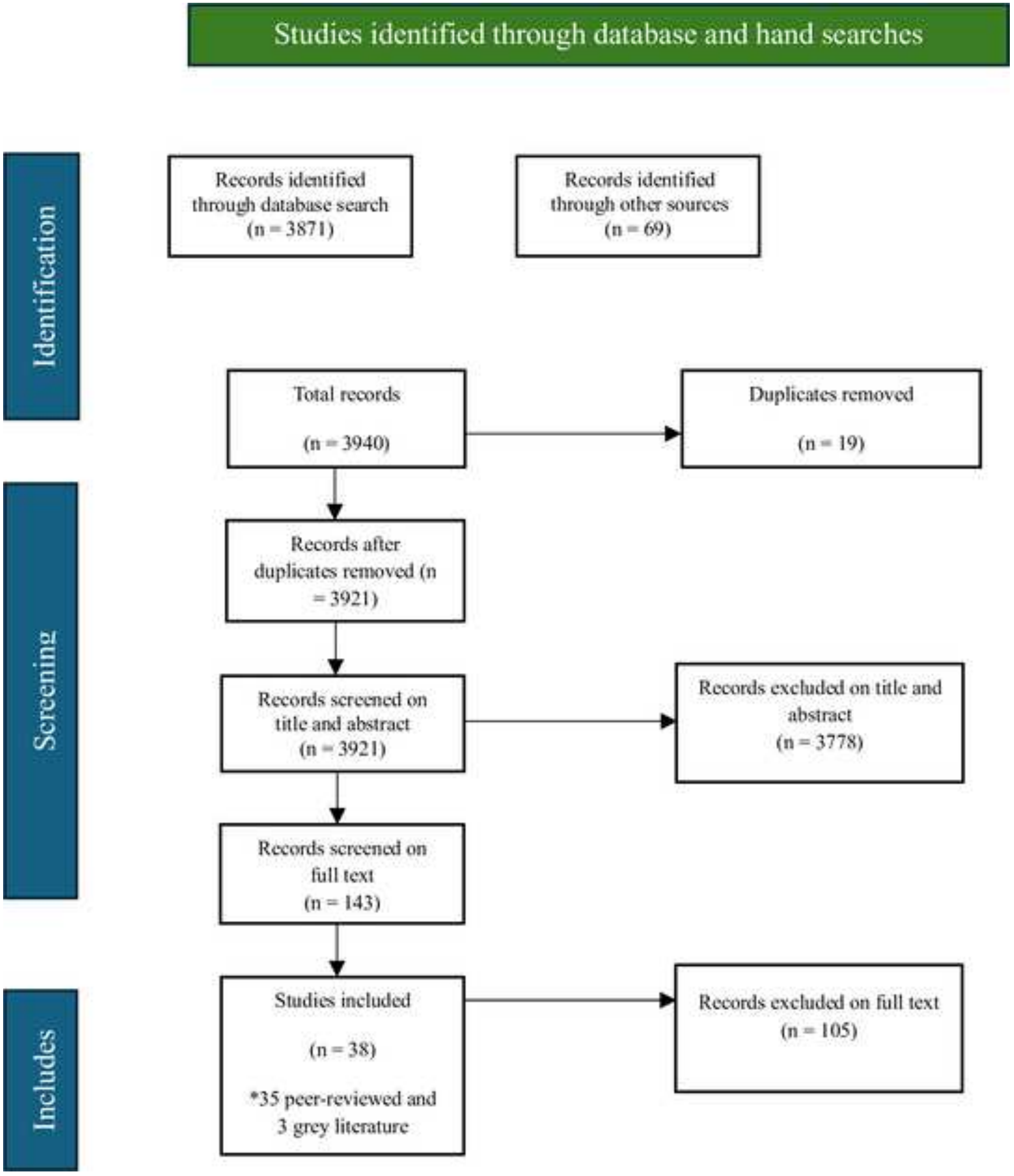

Figure 2 Click here to access/download;Figure;Fig 2.tif

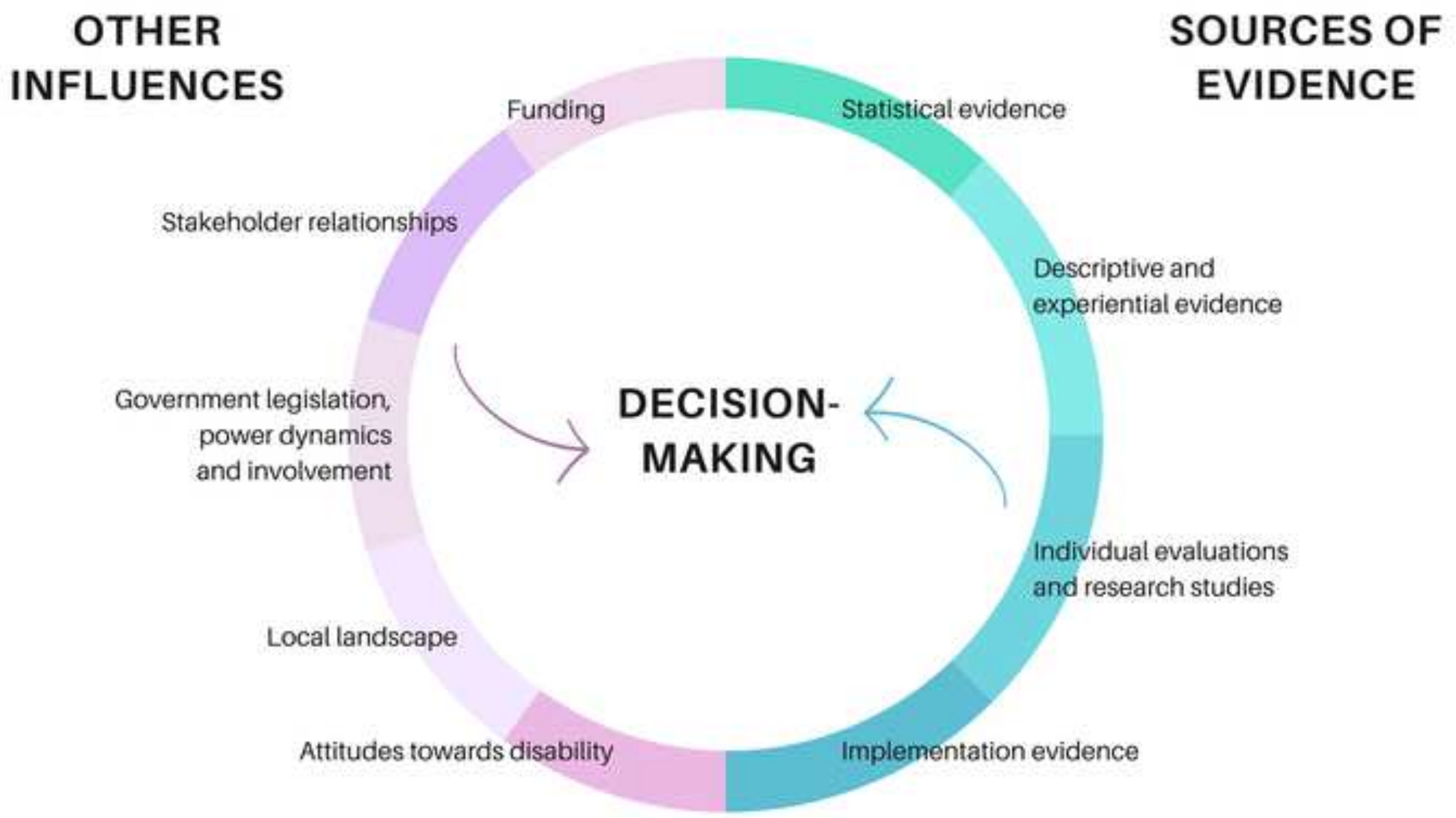

Figure 3

Click here to access/download;Figure;Fig 3.tif

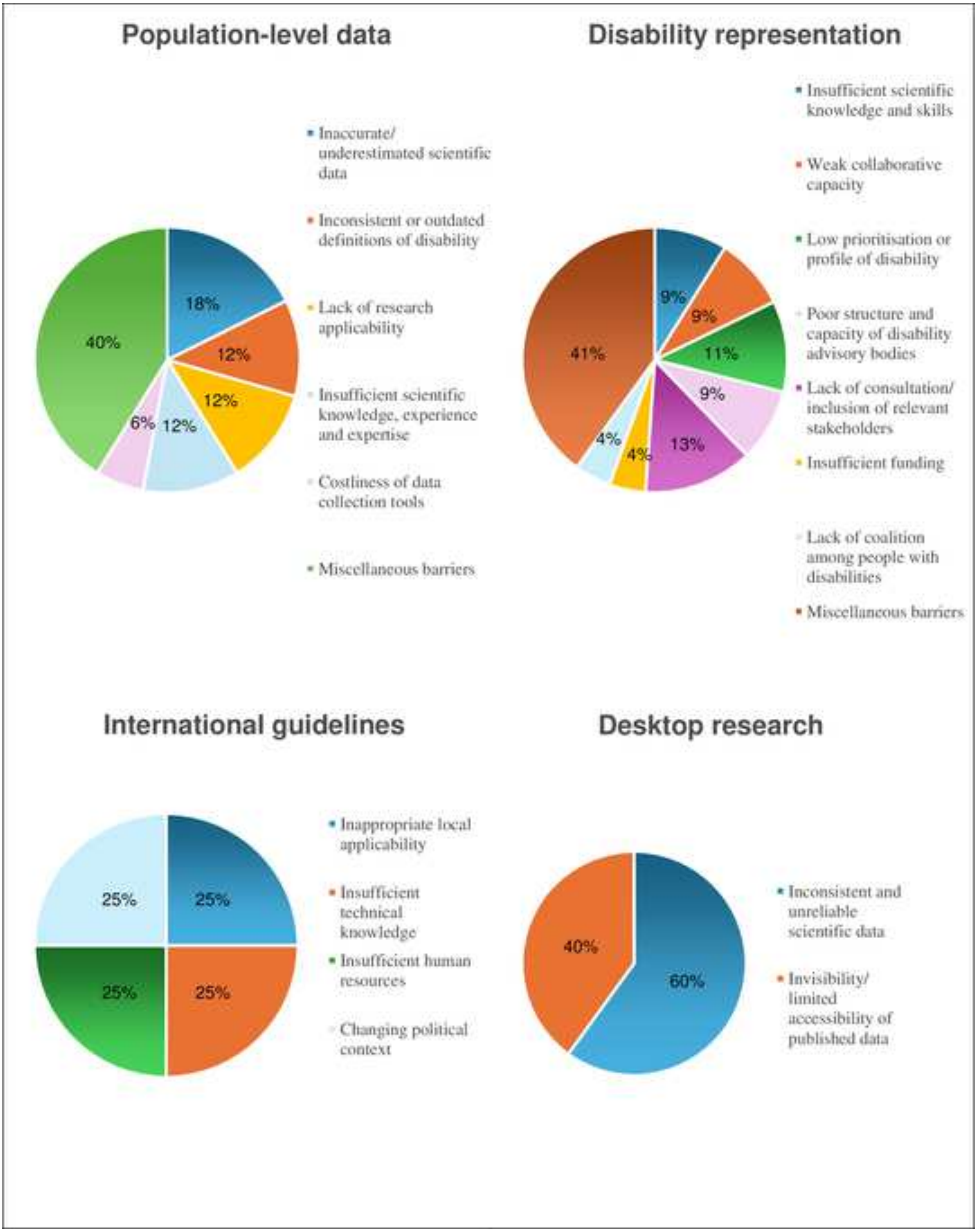

Figure 4 Click here to access/download;Figure;Fig 4.tif

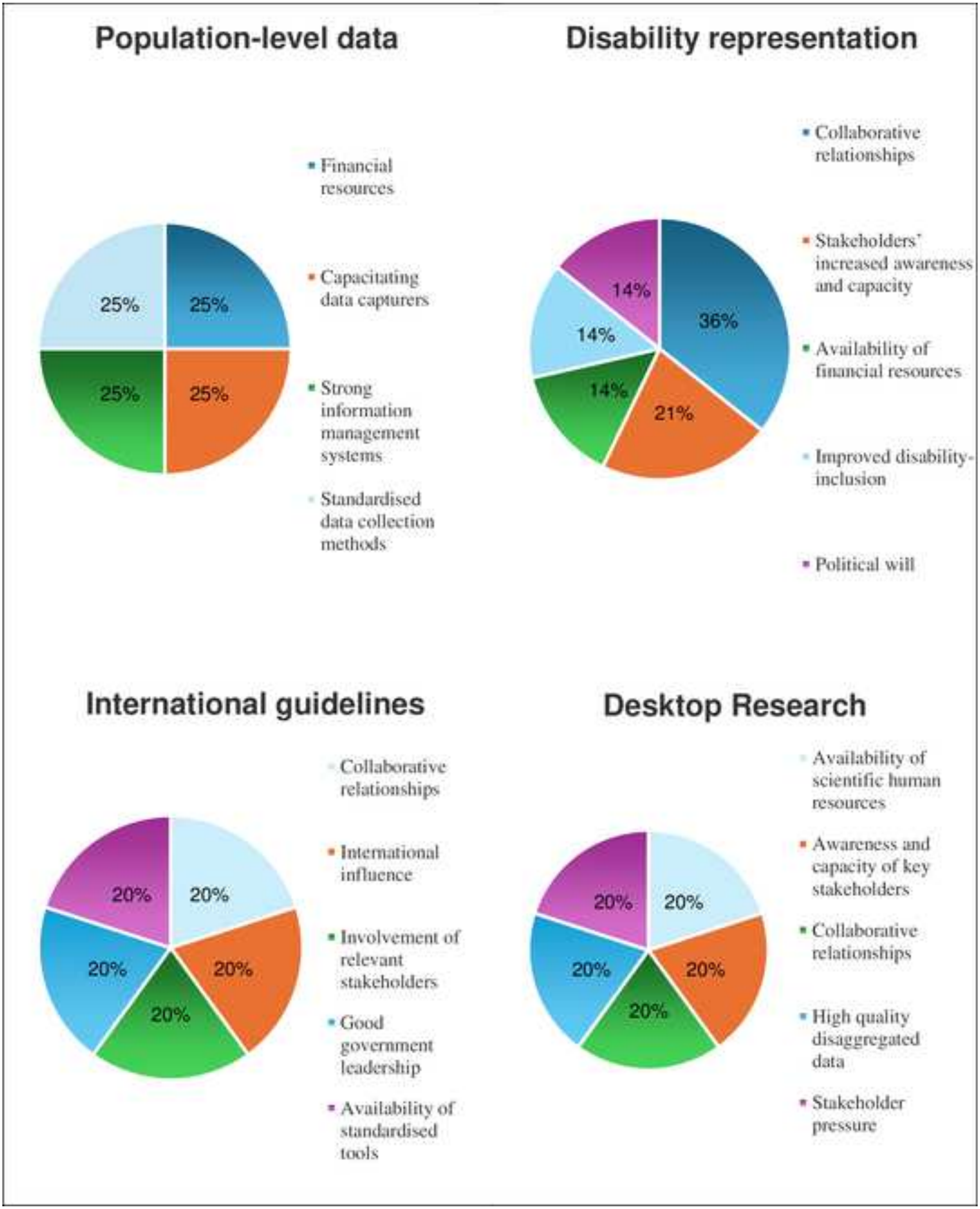

Figure 5

Click here to access/download;Figure;Fig 5.tif

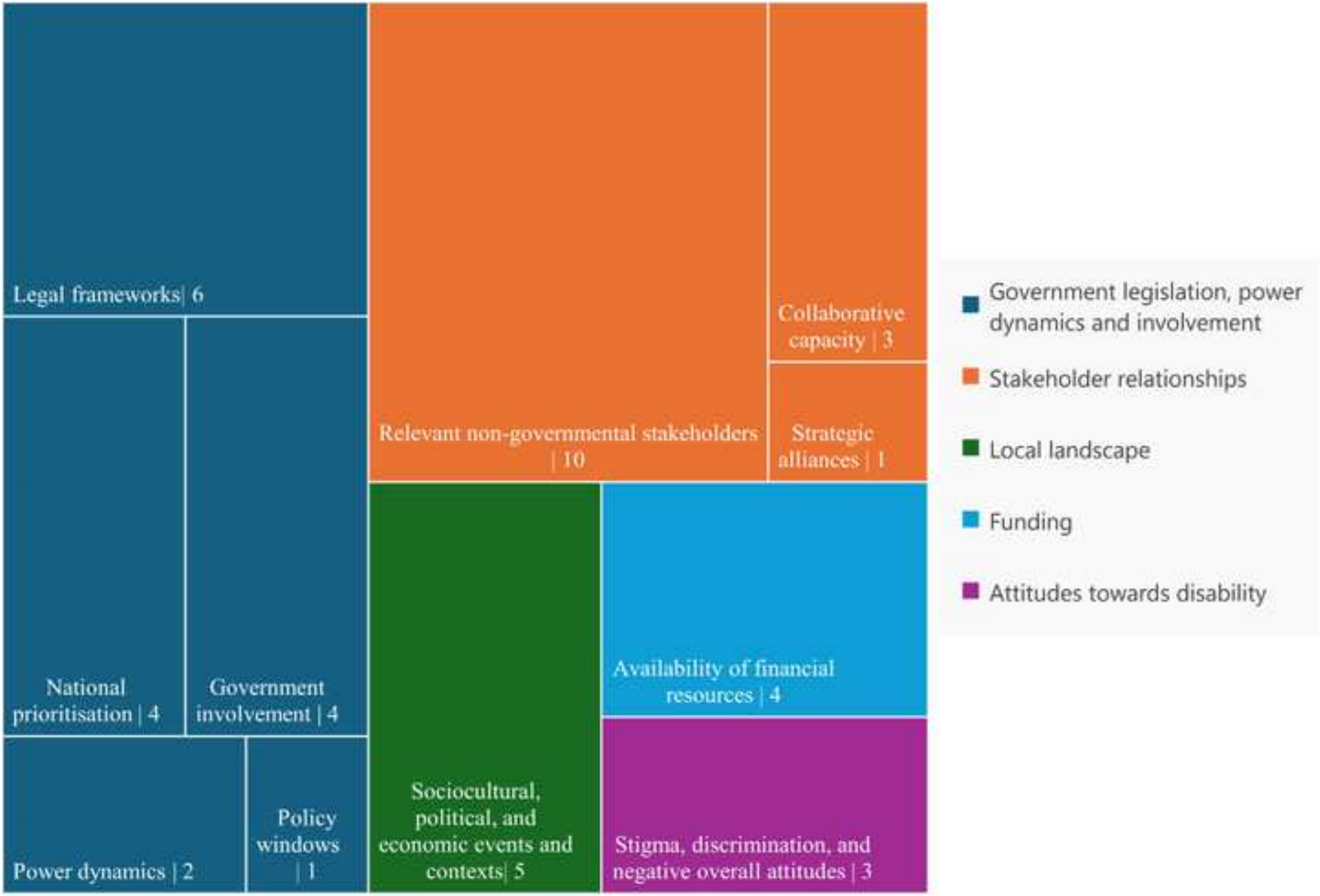

Click here to access/download
Supporting Information
S1 PRISMA-ScR-Checklist.pdf

Supporting Information 2

Click here to access/download
Supporting Information
S2 Search strategy.pdf
