# Supplementary material for: Sources of evidence, pathways, and processes to support disability-inclusive decision-making in low- and middle-income countries: A scoping review
Source: PLOS Glob Public Health. 2025 May 12;5(5):e0004555. doi: 10.1371/journal.pgph.0004555 (PMC12068629; doi:10.1371/journal.pgph.0004555)
Supplement: Attachment — Submitted filename: PGPH-D-24-02548_Comments_SN.docx [file pgph.0004555.s004.docx]

**Introduction**: The introduction is well-presenting the significance of this study. However, adding literature-based reasoning on which specific sectors, e.g., health, economy, and education, have mostly excluded evidence-based disability-inclusive decision-making and implementation and how this study will contribute to addressing that exclusion will strengthen the introduction.

**Method**: The method needs to be re-structured. It comprises all the required components but in a scattered and unnecessarily lengthy manner, making readers question its validity.

Authors should consider incorporating the PRISMA guideline in a flow chart form, which will include how many records were identified according to search strategy, how many were left after screening, how many were left after removing duplication, how many full articles were available and finally how many were included for analysis.

The search strategy should be very clear. What were the search words, and how were the words combined by using “AND” and “OR”? This should be explicitly mentioned in the search strategy table added in the supplementary file. However, the inclusion and exclusion criteria should be included in the method with justification. For example, if authors have not included any policy analysis in their study, then why?

The coding categories and their definitions are more suitable to present in the method section, not in the result.

**Results**: The authors have identified a wide range of themes from their in-depth analysis. However, the result section needs some re-organization to increase clarity and avoid unnecessary complexity.

In “Results Part I: The Literature as a Whole, Inclusive of Mental Health,” the sub-section ‘Types of sectors and topics covered’ can integrate types of literature. For example, how many were from the health sector? Then, the papers from the health sector can be categorized into quantitative, quantitative, and mixed methods, and the country-specific focus can be explained under quant, qual, or mixed methods. Though the country-specific focus is not very important, as the study revolves around LMIC’s activities

For the sub-sections ‘Statistical Evidence’, and ‘Descriptive and experiential evidence’ under “Sources of evidence and barriers and facilitators to use of evidence sources,” I will again suggest presenting the result sector-wise (education, health or multi-sectoral or solely individual or social welfare of people with disabilities) to improve clarity and maintain logical flow. Please try to shorten it, and I find some parts of the result more suitable for the discussion section.

**Discussion**: The discussion section is well-written, and a few comments are attached to the document. It needs to be adjusted with the re-structured result section.
Authors can look at the following papers by Dr Wilbur to strengthen their discussion;

1. Translating disability-inclusive WASH policies into practice: lessons learned from Bangladesh

2. A qualitative cross-sectional study exploring the implementation of disability-inclusive WASH policy commitments in Svay Reing and Kampong Chhnang Provinces, Cambodia

**Conclusion**: Authors should include a very brief summary of their significant findings/themes at the beginning of the conclusion and then highlight how this study contributes to paving the pathways and processes to support disability-inclusive decision-making in LMICs
