# Supplementary material for: Sources of evidence, pathways, and processes to support disability-inclusive decision-making in low- and middle-income countries: A scoping review
Source: PLOS Glob Public Health. 2025 May 12;5(5):e0004555. doi: 10.1371/journal.pgph.0004555 (PMC12068629; doi:10.1371/journal.pgph.0004555)
Supplement: Attachment — Submitted filename: Response to Reviewers.docx [file pgph.0004555.s008.docx]

Response letter

| **REVIEWER COMMENTS ENCLOSED IN MANUSCRIPT** | | |
| --- | --- | --- |
| **REVIEWER** | **COMMENTS** | **RESPONSE** |
|  | | |
| REVIEWER #1 | **Abstract** | |
|  | Lines 2-3: account for disability in what policy & programmes, and exclusions from what? | 1. On page 1, in lines 2-4, we have specified the types of policies and programmes, in terms of sectors, that fail to account for disability sufficiently, particularly in LMICs. |
|  | Line 5: within health systems? or intersectoral? | 2. In order to streamline the content of our manuscript and maintain the world limit for the abstract, we have edited this portion out. |
|  | **Introduction** | |
|  | Line 31: health services? or what services do you mean? | 3. The various services in question were in relation to each sector listed in lines 49-50 of page 3, previously line 31. |
|  | Line 34: Good to include a definition for disability and a conceptual view that guided the identification and inclusion of studies | 4. Thank you for the suggestion. We have now clarified this (please see line 303 on page 13). As is widely known, the definition of disability is a contested one. However, we draw from the definition of disability from the UNCRPD. Please see lines 175-178 on page 8. |
|  | Line 36: what about in global health? | 5. In lines 61-68 on page 3 and lines 116-129, we included a brief description of evidence-based decision-making in global health and global health’s focus in the context of disability. |
|  | Line 61: who attributes this? might require to be backed up by a reference | 6. On page 4, line 81, previously on line 61, we clarified the text and added the relevant sources. |
|  | Line 65-67: outline what types of information- health service use? health outcomes? types of disability? | 7. Lines 86-87, previously lines 65-67, now includes specific forms of information. |
|  | **Materials and methods** | |
|  | Lines 148-154: This can be moved to the results section- stating why you differentiate the categories there | 8. Due to our focus on disability-specific publications, please note that this section has been removed. |
|  | Line 185: This is not clear- is this people with disabilities and how policies in different sectors respond to their needs, or is this policymakers from the different sectors- please clarify and emphasise this in the introduction framing and methods section. | 9. The participant group referred to in line 254 on page 11, previously line 185, focused specifically on key stakeholders from different sectors who are involved in high-level decision-making. This was based on our inclusion criteria as now stated on pages 7-9, line 168-213, and introduction framing in lines 132-134; 136-137 on page 6. |
|  | **Results** | |
|  | Lines 230-233: Why was this decision made, and is this in line with a conceptual understanding/ definition of disability that you set out. It is not clear why mental health was removed from this stage when it was included in the prior stage. Please make a decision to include or exclude mental health across all sections- drawing on a conceptual definition you anchor on. | 10. Thank you for encouraging us to rethink our presentation of this. At the time of our formative work for this scoping review, we found that psychosocial impairments, often classified under mental health rather than disability, received more policy attention, investment, and publications than sensory, physical, intellectual, self-care, or communication impairments (e.g., in rehabilitation, assistive technology, or early identification). Therefore, we focused on disability literature while excluding psychosocial impairments/mental health. This does not imply that psychosocial disability is not a form of disability but reflects the challenge of distinguishing it from broader mental health literature, which we considered as a whole. We recognised that the concerns of people with sensory, physical, intellectual, self-care, or communication impairments may differ systematically from those in global mental health literature, limiting the latter’s applicability to disability more broadly. We explain this in more detail on lines 179-196 on pages 8 and have removed all content focusing on mental health topics in this manuscript. |
|  | Line 233-234: It is not clear why this sentence and exclusion is necessary- please clarify or remove | 11. Please see response point 8. |
|  | Line 240: This section can be condensed into a table for ease of reading and to shorten the paper | 12. According to Reviewer #1’s comments on lines, previously 240, 264 and 273, we have tabulated the sections and merged them as suggested in order to shorten the manuscript’s length. Please see table 2 from line 320 on pages 14-15. |
|  | Line 264: Again, can be made into a table for ease of reading and to shorten paper | 13. Please see response point 12. |
|  | Line 273: this section should be merged with the previous, and made into a table to shorten the paper | 14. Please see response point 12. |
|  | Line 278: Again, this decision to exclude mental health from this section is not clear- if it is excluded here, please remove from previous section and give a strong conceptual framing around why only other disabilities were included. It will still be a strong enough with or without mental health, but please be consistent. | 15. Please see response point 10. |
|  | Lines 283-288: These are your strong points in the finding- please collapse into a table and then spend some words describing these details. | 16. According to the reviewer’s comments, we have tabulated this section as suggested in order to shorten the manuscripts length. Please see Table 3 line 332 on pages 15 - 16. |
|  | Lines 298-301: Are these from the retrieved papers? if not it is a discussion point- please keep this section to only results, and discuss the points in the next. | 17. We have moved this into the “Methods and Materials” section introducing this is an approach we used to organise sources of evidence within the results section. Please see lines 277-297 on pages 12-13. |
|  | Line 303: Please look up in text citation conventions- year might be required. | 18. The year is not included for Vancouver referencing. |
|  | | |
| REVIEWER #2 | **Introduction** | |
|  | Lines 60-69: Without mentioning specific sectors (e.g. education, health, economy etc) it is difficult to grasp the justification of conducting this study. Please draw from the literature and interconnect these information with some sector-specific examples, where people with disabilities were excluded from evidence-based decision making process | 19. Specific sectors in question are found in lines 49-50 as an indication that this exclusion is true across all sectors. Furthermore, it is not about decision-making at an individual level or the public participation of people with disabilities, but rather, whether or not they are catered to in policies and programmes. |
|  | Lines 90-93: Is this the research question? | 20. No. We have modified this portion of text to reflect the intended meaning more accurately. Please see lines 130-134 on pages 6. |
|  | **Materials and Methods** | |
|  | Lines 100-114: If these are the research question, include these on introduction | 21. Please see lines 132-137 on page 6. Within the introduction, we have also added more detail to the text to ensure clarity of what our research question is exploring. |
|  | Line 119: Please list these five steps, so the readers can relate with the following section | 22. We have modified the text to include these 5 steps (please see lines 163-167 on page 7). |
|  | Lines 132-133: Please clearly explain what were the inclusion and exclusion criteria | 23. The inclusion and exclusion criteria were previously listed in lines 215-220 on pages 8 and 9. These have been moved up to the “Data sources and search strategy” section. Please see lines 169-213 on pages 8 and 9. |
|  | **Results** | |
|  | Line 213: Authors need to mention the numbers here. It should be clearly written how many records in total were screened and how many duplicates were removed. | 24. We have added the exact number of records screened and duplicates removed. Please see lines 305 and 304 on page 13, respectively. |
|  | Lines 233-234: Authors need a give a brief explanation for this. Because works of academicians can and do influence policy making and implementation, even in LMICs. | 25. Though academics may be influential in informing, supporting, or challenging NGO or government decision-making, they rarely have the power to make final decisions concerning policy development and programming. We have added this justification to the text. Please see lines 316-318 on pages 13. |
|  | Lines 243-244: This sentence is difficult to understand, please clarify. | 26. This sentence has been removed. We have tabulated this to simplify it. Please see Table 2 on pages 14-15. |
|  | Lines 249-252: Both In-depth interviews and Focus Group Discussion are tools used in qualitative research. That's why it will be better to incorporate these under qualitative methods. And also briefly mention about the tools used in quantitative papers | 27. The reason for reporting this result was to highlight the overall most common method used across all included papers, followed by identifying the specific data collection approach used. |
|  | Line 288: Please provide some examples for clarification. Was there mention of any act that supports social welfare of people with disabilities? | 28. N/A. This can now be found condensed in Table 3, on pages 15-16. |
|  | Lines 386-387: This should not be explained here, but elaborated in discussion section with an attempt to answer the 'why' those evidences were not perceived as relevant in inclusive education efforts | 29. Now found on line 397-398, the text in reference does not serve as an explanation meant to discuss the perceived relevance of this form of evidence by us as the manuscript’s authors, but was merely a report of the results itself from the paper included in this scoping review. |
|  | Line 489: First? does this mean this 'other influence' was the most prominent one? please explain | 30. This was describing the most frequently occurring influence noted across the literature. We had ranked these influences in terms of frequency, and not impact, though “prominence” could be inferred due to its impact in different, locations, contexts and among various actors. However, the latter was not our approach. |
|  | Lines 509-511: This needs to be explained in discussion section | 31. We have moved this sentence down to the discussion section. Please see lines 656-658 on page 29. |
|  | Line 584: Stigma and disability discrimination can be experienced in every sector, not only in education. However, if the evidence form the literature indicates this discrimination experienced only for learning and education, then it can be moved to discussion section as one of the underlying barriers of evidence-based disability inclusive policy making in education | Agreed. However, our current results concerning stigma and disability discrimination reflect what the included papers, yielded from our search, reported on. Please see data repository containing our findings. We choose to keep it in the results so that we are able to bring it into our discussion as it is not just supporting literature, but part of our paper’s findings. |
|  | **Discussion** | |
|  | Lines 611-616: This finding was not explicitly presented in the result section. Authors should make sure new findings are not introduced in discussion and whatever they have discussed are clearly included in the result section | 32. This is in reference to the findings in Table 2 (line 322), page 14-15 concerning the most frequently occurring sector, and is not a finding itself. It is meant to correlate with participants’ prevailing beliefs who are found within this category (the health sector). |
| **REVIEW COMMENTS TO THE AUTHOR** | | |
| **REVIEWER** | **COMMENTS** | **RESPONSE** |
| REVIEWER #1: | Thank you for the opportunity to review this interesting manuscript. You have presented rich findings that will be useful for applied work in the future. There are comments to address below. Of major thought is consideration on the continued inclusion of the mental health studies. As I recommend below, I suggest you consider including findings from these or extracting into a second paper. They are lost in this paper. Good luck with the amendments and look forward to seeing this at next draft. Additionally, I note that your search was conducted nearly a year ago. I suggest you re-run the search for this year and include any published in 2024. Considering this won't be published by the journal until 2025, your search will be out of date quickly. It shouldn't be a lot of work to add in updated search results. | 33. We thank the reviewer for the compliment. Please see response point 10. |
|  | **Abstract** | |
|  | Include the databases searched | 34. We considered this, however, given the extensive literature search we did across several databases, we thought it best to keep the abstract as succinct as possible due to prescribed word limit. More details on the databases searched can be found on pages 9-10 within the “Materials and methods” section, for readers. |
|  | **Introduction** | |
|  | Line 36: Check journal guidance but may need year after ‘Goldman and Pabari’. | 35. The “year” is not included for Vancouver in-text referencing. |
|  | Line 37: You may need to explain what each of these steps include. I don’t intuitively know what ‘intervention selection’ is, for example | 36. We have expanded on the description as provided by the authors Goldman and Pabari. Please see lines 56-59 on page 3. |
|  | Line 47: This section on the what we mean by evidence is quite long-winded. If you are not addressing this in this study, as you say, then consider removing or condensing to very simple terms. From line 52 to 58 is particularly long-winded and could be refined | 37. We have condensed this section for a more concise explanation. Please see lines 73-80 on page 4. |
|  | Line 60: Need to spell out LMIC in full first | 38. We have added the full term to the text. (Please see line 65 on page 3). |
|  | Line 65: When describing the lack of disability data, be clearer with what you mean by “insufficient and irregular” | 39. We have clarified this within the text. (Please see lines (88-90) on pages 4). |
|  | **Materials and methods** | |
|  | Line 119: What do you mean a scoping review methodology? Outline from who and where this methodology has derived. And outline the five steps | 40. Please see response point 22. |
|  | You have not included a clear inclusion criteria. Please add using a clear structure, e.g. PICO or SPIDER. Include as a separate section before the search strategy. Note: I now see something akin to this at the start of the results. It should come in the methods and be more clearly structured, as suggested | 41. Thank you. We have amended the manuscript to reflect a clearer inclusion and exclusion criteria using the SPIDER framework. Please see response point 23. |
|  | Good array of databases used | 42. We thank the reviewer for this compliment. |
|  | The search for grey literature could be clearer. For each of these websites, how did you search for documentation? Did you search every page or use their search function? Why did you pick these organisations? Suggest also including the full names of organisations, not acronyms | 43. We have detailed our grey literature search strategies. Please see lines 235-238 on page 10.   The databases have been spelled out in full (lines 217-229 on page 9-10). |
|  | For the Google Scholar search, how did you ensure this was systematic? What search terms did you use? How did you know when you stop looking (i.e. did you look at the first 10 pages, for example)? | 44. We have detailed our Google Scholar search strategies. Please see lines 235-238 on page 10. |
|  | The search strategy looks fine, although you have not included in the search the names of actual countries classified as LMIC. This should be listed as a limitation, as you may have missed some studies that did not mention the search terms used, and simply named the country, e.g. Zambia without mentioning Africa or lmic or developing | 45. We understand the concern of the reviewers regarding the use of search terms such as Africa/LMIC/developing etc. without further specifying the search by country name. However, this did not affect the results yielded as papers with country names specified had appeared on our list. This can be attributed to researchers’ inclusion of keywords and MeSH terms in their articles that make it possible to retrieve them regardless (please see lines #77 and #85 of S1). |
|  | Line 133: “Worked in one pair to screen the literature”. You can likely delete this here as you explain in the study selection process. I had originally asked for more information here, but saw it came later, so suggest removing at this point, so as not to confuse the reader | 46. This portion of the sentence has been removed from the sentence on line 233 to avoid confusion and repetition. |
|  | Why did you use the cutoff of 1990? | 47. We have elaborated on the cut off on page 9 in lines 211-213. |
|  | Line 135: Your search was conducted nearly a year ago. Consider updating this search to capture data from 2024. This is important, given the increase in disability-related research we are seeing | 48. A full scoping review update is likely unnecessary as significant new research on disability-related decision-making in LMICs is unlikely to have emerged within a year. Possible delays for peer-reviewed publications, particularly in LMICs, mean that relevant studies may still be in preprint or under review, limiting their availability. Our scoping review is meant to be a broad overview rather than a frequently updated synthesis, and updates will be considered as necessary in cases where major policy changes or a substantial increase in research output has occurred.   Given the significant time and resource investment required, re-running the review may not be doable unless the journal is able to provide funding or support. Should the journal still see it necessary for a search to take place, we feel that a more efficient approach would be conducting a rapid review to capture any recent developments. |
|  | Line 157: Make clear by ‘below’ you mean ‘in the results’. I was unclear where Part 1 would be | 49. Please see response point 8. |
|  | **Results** | |
|  | In the PRISMA chart, I would expect to see reasons for exclusion at full-text in the relevant box | 50. Please see amendments made to PRISMA diagram (Fig 1). |
|  | Line 298: Add instead to the methods section under data analysis/synthesis as this is not results. Similarly, suggest outlining the details of each category in the methods, rather than at the beginning of each section in the results. The results feel long as is | 51. As per Reviewer 1 suggestions, we have moved this into the “Methods and Materials” section. Please see response point 17. |
|  | Line 331: You have already outlined that you are reviewing the literature for barriers (see line 310). Suggest review of results and methods to cut down on repetition | 52. We have removed this sentence to avoid repetition and streamlined the results section as much as possible. |
|  | The results are informative and well-structured. | 53. We appreciate the reviewer’s compliment. |
|  | Although I understand your rationale, it does seem a bit off in practice when you have not synthesised any information from the included mental health literature. The results are already very long and I am reluctant to add more, but I do think you need to consider providing a short paragraph on evidence related to mental health studies, under each evidence section. Otherwise why include them at all, except to show that mental health is a more researched area? Feels a wasted opportunity, given the effort to source the studies. If not here, consider providing in a separate paper and excluding from this. You make this note in the methods, to say “The concerns of people with sensory, physical, intellectual, self-care, or communication impairments may differ, in some systematic ways, from those documented in the global mental health literature, and so lessons learned from the latter literature may have limited applicability to the concerns of people with disabilities generally.” Perhaps then exclude them from this paper with this rationale and write another specific to mental health. | 54. Please see response point 10. |
|  | This is a long section and long piece of work generally. I encourage you to consider putting the table or some figures in as supplementary information | 55. We have modified manuscript as per the reviewer's suggestion. Table 1 is now supplementary information (Please see S4). |
|  | **Discussion** | |
|  | Generally a thoughtful and interesting discussion | 56. We appreciate the reviewer’s compliment. |
|  | Line 681: Could you structure this as a clearer recommendations section and consider bulleting the different recommendations | 57. Thank you. We appreciate the suggestion but prefer to keep our recommendation section in a paragraph format. |
|  | Limitations: See notes in the methods section | 58. These have been addressed in previous section. |
|  | | |
| REVIEWER #2: | **Introduction** | |
|  | The introduction is well-presenting the significance of this study. However, adding literature-based reasoning on which specific sectors, e.g., health, economy, and education, have mostly excluded evidence-based disability-inclusive decision-making and implementation and how this study will contribute to addressing that exclusion will strengthen the introduction. | 59. We thank the reviewer for this compliment. Kindly refer to response point 19 and lines 134-138, respectively, concerning literature-based reasoning and how we describe our study’s contribution to addressing that exclusion. |
|  | **Method** | |
|  | The method needs to be re-structured. It comprises all the required components but in a scattered and unnecessarily lengthy manner, making readers question its validity. | 60. We have modified this section as per the reviewer’s earlier suggestions. Please see response points 12, 17, 23, 41, 52 and 55. |
|  | Authors should consider incorporating the PRISMA guideline in a flow chart form, which will include how many records were identified according to search strategy, how many were left after screening, how many were left after removing duplication, how many full articles were available and finally how many were included for analysis. | 61. Thank you, reviewer. We have already done this. Please see Fig1.tif. |
|  | The search strategy should be very clear. What were the search words, and how were the words combined by using “AND” and “OR”? This should be explicitly mentioned in the search strategy table added in the supplementary file. However, the inclusion and exclusion criteria should be included in the method with justification. For example, if authors have not included any policy analysis in their study, then why? | 62. Yes, we have already included it in our S2 file. Kindly refer to it for more information on our search strategy. Furthermore, we have also moved our inclusion and exclusion criteria into the “Methods and Materials” section. Please see response point 23. |
|  | The coding categories and their definitions are more suitable to present in the method section, not in the result. | 63. Kindly refer to response point 52. |
|  | **Results** | |
|  | The authors have identified a wide range of themes from their in-depth analysis. However, the result section needs some re-organization to increase clarity and avoid unnecessary complexity. | 64. We thank the reviewers for sharing this observation. We have modified the results section to reflect this. Kindly refer to Results section from page 12 of the manuscript. |
|  | In “Results Part I: The Literature as a Whole, Inclusive of Mental Health,” the sub-section ‘Types of sectors and topics covered’ can integrate types of literature. For example, how many were from the health sector? Then, the papers from the health sector can be categorized into quantitative, quantitative, and mixed methods, and the country-specific focus can be explained under quant, qual, or mixed methods. Though the country-specific focus is not very important, as the study revolves around LMIC’s activities. | 65. Please see response number 12. |
|  | For the sub-sections ‘Statistical Evidence’, and ‘Descriptive and experiential evidence’ under “Sources of evidence and barriers and facilitators to use of evidence sources,” I will again suggest presenting the result sector-wise (education, health or multi-sectoral or solely individual or social welfare of people with disabilities) to improve clarity and maintain logical flow. Please try to shorten it, and I find some parts of the result more suitable for the discussion section. | 66. Thank you for the suggestion. We have shortened this section of our results. However, we are unable to break the sub-sections down in the way Reviewer 2 suggested. This is due to the overlap in sectors as study samples in several of the records included are samples that consist of more than just one sector (i.e., the presence of health, education and social development sectors, etc. in a study sample). |
|  | **Discussion** | |
|  | The discussion section is well-written, and a few comments are attached to the document. It needs to be adjusted with the re-structured result section. Authors can look at the following papers by Dr Wilbur to strengthen their discussion;  1. Translating disability-inclusive WASH policies into practice: lessons learned from Bangladesh  2. A qualitative cross-sectional study exploring the implementation of disability-inclusive WASH policy commitments in Svay Reing and Kampong Chhnang Provinces, Cambodia | 67. We thank the reviewer for sharing this suggestion. |
|  | **Conclusion** | |
|  | Authors should include a very brief summary of their significant findings/themes at the beginning of the conclusion and then highlight how this study contributes to paving the pathways and processes to support disability-inclusive decision-making in LMICs | 68. We have amended the conclusion section as suggested. Please see conclusion section on page 31. |
|  | | |
| REVIEWER #3: | 1. This is a clear and well-written manuscript and of great relevance in relation to the neglected issue of disability inclusion in low- and middle-income country (LMIC) policies and programmes. It addresses an important knowledge gap and helpfully unpacks the piecemeal rather than systematic way in which policies and programmes are shaped, both because of a dearth of LMIC evidence and because where evidence does exist it may not be considered suitable or may not be drawn upon in a systematic way. Although this is not a central focus of the study, the authors also note that what constitutes evidence is itself not fully articulated, and that in the case of LMICs even basic data such as demographics and specific disability information tends to be highly lacking, compared with high-income countries (HICs). Thus, they acknowledge that evidence-based decision-making models developed in HICs may thus be difficult to apply in LMICs. | 69. We thank the reviewer for the observation as well as compliments paid. |
|  | 2. The authors have dealt carefully with the preponderance of mental health literature and provide a justification for how they did so. They provide a clear and reader-friendly description of their study selection, data extraction and data analysis processes, helpful to potential future researchers and creating a strong sense of trustworthiness and credibility. | 70. We thank the reviewer for the observation as well as compliments paid. |
|  | 3. Table 1 gives a clear and thorough overview of the selected studies but I suggest that in lines 228-230 mention is made of the first 16 studies (with a core focus on disability) being shown in green and the last 22 (with a core focus on mental health) being shown in blue. With this having not been mentioned overtly, and due to the studies not being numbered, the blue took me by surprise and caused me to first look back at the text for clarification and then count the green studies to sure that the colours had been applied in the way I surmised. | 71. Table 1 has been removed from the manuscript and formatted into a supplementary file (S4). This has been replaced by Table 2. Please see response point 12 for further details. |
|  | 4. The results section is well set out, clear to follow and provides additional trustworthiness elements such as explanations of how coding categories were developed and defined. Figures 2, 3, 4 and 5 are bold and succinct, but I do note that the use of colour can be problematic both for people with colour blindness (ironic, given the focus of the paper) and for reproduction of the article in black and white. The latter is unfortunately likely to happen in lower-resourced settings, reducing its value to stakeholders like policymakers and civil society organisations. | 72. The authors thank the reviewers for this compliment. We have modified the figures to reflect this (Please see Figs. 1-5). |
|  | 5. The discussion section flows well, integrating the results and framing them against the literature before making some pertinent recommendations. | 73. We thank the reviewer for this compliment. |
|  | 6. The conclusion is somewhat long but does not suffer from repetition, and making it more concise is a nice-to-have rather than must-have. | 74. Thank you. We have streamlined the conclusion. |
|  | 7. There are a few language and typographical issues to address: a) In lines 265 and 267 I looked twice at the terminology "worked with", which seems strange - it should perhaps be replaced with something like "focused on", to more aptly reflect the authors' roles in the relevant studies/sources.  b) In line 283 there is sudden use of a fractional percentage ("8.50%") whereas for the most part whole numbers are used and I presume the authors thus rounded off decimal points. For reader-friendliness, I suggest applying whole numbers consistently throughout the manuscript.  c) Although I spotted almost no typos, one in line 456 (in the word "insufficient") did jump out at me. | 75. a) As per Reviewer #1’s earlier suggestion, we have formatted this text into a table for conciseness. Kindly see Table 2 on page 14-15.   b) We thank the reviewer for this observation. Please see changes made to line 339, previously line 283, on page 16. We have also rounded off percentages to the nearest whole number in lines 426 and 445.  c) Thank you. Previously in line 456, please see typo corrected in line 452 on page 21. |
|  | 8. Finally, I have one big concern and that is the reference list. Too many references fail to provide enough information for them to be reliably located by readers (eg: some do not provide the name of the institution/organisation which produced them, and many do not provide a URL even though one is almost certainly available and could have been readily found and included by the authors, as a service to their readership). Particular attention needs to be paid to refs 1, 4, 8, 13, 15, 21, 29 and 46 in this regard, and it is especially important to ensure that the grey literature is findable. Ref 8 would benefit from adding capitals (Global Governance Programme & School of Transnational Governance) and should specify where this school is located. Ref 15 does not indicate which organisation produced that study/source, while ref 29 gives neither that sort of detail nor provides the source's date. Fixing up the reference list will make it match the generally impeccable writing style, attention to detail and reader-friendliness of the rest of the manuscript. | 76. We have updated the references according to the Vancouver referencing style. Please see references 1, 4, 8, 13 (now ref 20), 15 (now ref 22), 18 (now ref 24), 21 (now ref 14), 23 (now ref 33), 29 (now ref 38), 46 (now ref 55), and ref 56. |
| **ADDITIONAL EDITOR COMMENTS** | | |
| **COMMENTS** | | **RESPONSE** |
| Thank you for submitting this important work for publication consideration. This work has been reviewed by three reviewers who have provided constructive feedback to strengthen the manuscript. I am recommending a major revision, for the reason that some conceptual details and related methods need to be clarified, along side an overall review of the writing- which feels rather long-winded at the moment. Readers are time-poor, and often need to be pointed towards the important points quickly. Some of your important points are buried in text, and not signposted as the important findings. There are several areas particularly in results section 1 that can be collapsed into tab  les, and words can be better used to discuss rather than describe. | | 77. We thank the reviewers for sharing their insight and thoughtful contributions. We have edited the document, particularly section 1 in order to shorten the manuscript length, and sign-posted the important findings. |
| Please consider the feedback from reviewers carefully and respond to this in your resubmission. Importantly, please review the reference list and check missing details, refer to PRISMA diagram, search strategy and inclusion details more clearly in methods, and re-run the search in line with R1 suggestion to update results. This also aligns with the point around mental health inclusion below. | | 79. We have modified the text to reflect these suggestions in response points addressing these comments earlier in the table. |
| Additionally, I also have reviewed the paper and annotated some questions and points to clarify as comments in the attached PDF. My main concerns for this work are the framing around global health literature and scholarship, and then the inclusion/ exclusion of mental health in the overall analysis. | | 80. We have modified the text to reflect these suggestions in response points addressing these comments above, within the table. |
| 1) This is a global health journal, so readers will expect to see some framing around related topics and conversations. It is not clear at the outset if you refer to disability policy beyond the health system- this is an important framing bit that will strengthen the paper. You have included policy outside health which is very pertinent to intersectoral involvement and engagement by health system actors. Once you shorten and tighten the overall writing, please consider including a framing around the importance of intersectoral coordination, collaboration etc- and how that can benefit the goals of global health actors, particularly around disability and health policy. Additionally, in the discussions, please consider reflecting on what health sector actors can learn from the non-health sector stakeholders- as described in the included papers, and how this can also serve intersectoral collaboration work in global health. | | 81. Thank you for this observation. We have amended the transcript to include a global health framing. Please see response point 5 and lines 611-617, 620-624 and 632-635 on pages 27-28. |
| 2) The inclusion and exclusion of mental health policies is rather confusing. It seems like a matter of convenience rather than a conceptual decision. My suggestion is to either include mental health across all sections of analyses or remove it entirely. The paper will still serve its intended goals with or without mental health inclusion. You can anchor strongly on a conceptual definition of disability, and how disability is often framed in policy within LMICs- you could describe this in the introduction to guide readers. If you decide to remove mental health, you could say something like- in this paper we focus on the case of physical disabilities (for example- please clarify what you include, why this supports the research aim). | | 82. Please see response point 10. |
