# Supplementary material for: Sources of evidence, pathways, and processes to support disability-inclusive decision-making in low- and middle-income countries: A scoping review
Source: PLOS Glob Public Health. 2025 May 12;5(5):e0004555. doi: 10.1371/journal.pgph.0004555 (PMC12068629; doi:10.1371/journal.pgph.0004555)
Supplement: Attachment — Submitted filename: Response to Reviewers_Apr_2025.docx [file pgph.0004555.s009.docx]

| **Editor comments** | **Responses** |
| --- | --- |
| 1. Requesting a more prominent framing and specific mention of global health (instead of international development) in the introduction, with relevant global health references. | We have rearranged some of the content in our introduction and added the relevant references to make our global health framing more apparent. Please see lines 55 to 71. This framing and reference point is also used in the Discussion. |
| 2. Please clarify and state if you are studying intersectoral disability policy and link this back to how this is crucial for global health and the health outcomes of people with disabilities in the introduction. | We have further clarified this in lines 61 to 71. |

**Response Table**
